# Human Circulating Antibody-Producing B Cell as a Predictive Measure of Mucosal Immunity to Poliovirus

**DOI:** 10.1371/journal.pone.0146010

**Published:** 2016-01-05

**Authors:** Ayan Dey, Natalie A. Molodecky, Harish Verma, Prashant Sharma, Jae Seung Yang, Giulietta Saletti, Mohammad Ahmad, Sunil K. Bahl, Thomas F. Wierzba, Ranjan K. Nandy, Jagadish M. Deshpande, Roland W. Sutter, Cecil Czerkinsky

**Affiliations:** 1 International Vaccine Institute, Seoul, South Korea; 2 World Health Organization, Geneva, Switzerland; 3 Department of Microbiology and Immunology, Seoul National University College of Medicine, Seoul, Republic of Korea; 4 World Health Organization- National Polio Surveillance Project, New Dehli, India; 5 Vaccine Development Global Program, PATH, Washington, DC, United States of America; 6 National institute of Cholera and Enteric Diseases, Kolkata, India; 7 Enterovirus Research Centre, Mumbai, India; 8 Institut de Pharmacologie Moleculaire et Cellulaire, CNRS-INSERM-University of Nice-Sophia Antipolis, Valbonne, France; New York State Dept. Health, UNITED STATES

## Abstract

**Background:**

The “gold standard” for assessing mucosal immunity after vaccination with poliovirus vaccines consists in measuring virus excretion in stool after challenge with oral poliovirus vaccine (OPV). This testing is time and resource intensive, and development of alternative methods is a priority for accelerating polio eradication. We therefore evaluated circulating antibody-secreting cells (ASCs) as a potential means to evaluate mucosal immunity to poliovirus vaccine.

**Methods:**

199 subjects, aged 10 years, and previously immunized repeatedly with OPV, were selected. Subjects were assigned to receive either a booster dose of inactivated poliovirus vaccine (IPV), bivalent OPV (bOPV), or no vaccine. Using a micro-modified whole blood-based ELISPOT assay designed for field setting, circulating poliovirus type-specific IgA- and IgG-ASCs, including gut homing α4β7+ ASCs, were enumerated on days 0 and 7 after booster immunization. In addition, serum samples collected on days 0, 28 and 56 were tested for neutralizing antibody titers against poliovirus types 1, 2, and 3. Stool specimens were collected on day 28 (day of bOPV challenge), and on days 31, 35 and 42 and processed for poliovirus isolation.

**Results:**

An IPV dose elicited blood IgA- and IgG-ASC responses in 84.8 to 94.9% of subjects, respectively. In comparison, a bOPV dose evoked corresponding blood ASC responses in 20.0 to 48.6% of subjects. A significant association was found between IgA- and IgG-ASC responses and serum neutralizing antibody titers for poliovirus type 1, 2, 3 (p<0.001). In the IPV group, α4β7^+^ ASCs accounted for a substantial proportion of IgA-ASCs and the proportion of subjects with a positive α4β7^+^ IgA-ASC response to poliovirus types 1, 2 and 3 was 62.7%, 89.8% and 45.8%, respectively. A significant association was observed between virus excretion and α4β7^+^ IgA^-^ and/or IgG-ASC responses to poliovirus type 3 among immunized children; however, only a weak association was found for type 1 poliovirus.

**Discussion:**

Our results suggest that virus-specific blood ASCs, especially for type 3 poliovirus, can serve as surrogate of mucosal immunity after vaccination. Further studies are needed to evaluate the duration of such memory responses and to assess the programmatic utility of this whole blood-based mucosal ASC testing for the polio eradication program.

## Introduction

Since the world committed to eradicating poliomyelitis in 1988, there has been great progress with over 99% decrease in global polio cases. As of May 2015, three countries remain endemic to poliovirus transmission—Nigeria, Pakistan and Afghanistan [1]. Immune protection to poliomyelitis comes in two forms—humoral and mucosal. Humoral immunity protects from paralytic poliomyelitis and protection against disease correlates with induction of serum poliovirus-neutralizing antibody [2, 3]. Humoral immunity, however, does not prevent person-to-person transmission of poliovirus. Halting transmission of poliovirus is essential for global eradication of the disease. Mucosal immunity is assumed to protect against poliovirus entry into and transmission from the intestinal and nasopharyngeal mucosae, the primary sites of poliovirus replication, thereby halting person-to-person transmission of infectious virions.

The gold standard for determining poliovirus-specific mucosal protection is measuring excretion of virus in stool samples following a challenge dose of OPV. Absence of or reduced shedding is an indicator of mucosal intestinal protection. However, measuring virus excretion in stools following OPV challenge is both time and resource intensive. Alternative methods for assessing mucosal immunity have been explored including measurement of poliovirus-specific antibodies in mucosal excretions/secretions such as feces, nasopharyngeal swabs, breast milk and saliva [4–6]. To date, none of these methods have gained general acceptance as mucosal correlates (or even surrogates) of immune protection against poliovirus transmission. Although secretory IgA (sIgA) is by and large the predominant class of Ig in humans and especially in mucosal tissues [7], protective levels of sIgA antibodies against poliovirus replication are unknown, and correlations between sIgA antibody levels and poliovirus shedding have not been consistently observed [4, 8]. Hence, a standardized assay for measuring poliovirus-specific mucosal IgA antibodies has yet to be discovered. In the absence of a standardized assay, formal proof of the role if any of such antibodies in intestinal and/or pharyngeal protection against poliovirus has remained elusive.

In addition to directly measuring specific antibodies in external secretions, secretory immunity can be assessed by measuring circulating antigen-specific ASCs expressing mucosal homing receptors [5, 9]. Blood ASCs are plasma blasts, the immediate precursors of tissue plasma cells, the primary effector component of the adaptive humoral response to foreign antigens [10–12]. Upon re-exposure to antigen, a subpopulation of ASCs migrates to effector lymphoid tissues and can be detected transiently in peripheral blood [13]. Therefore, the detection of ASCs in blood provides an early indication of recent or persistent exposure to foreign antigens in peripheral as well as in mucosal tissues [13, 14]. Furthermore, ASC precursor B cells activated at mucosal sites coordinately express tissue-specific homing receptors and chemokine receptors which direct their selective migration to specific mucosal tissues [15, 16]. The integrin α4β7 is an important receptor that mediates trafficking of lymphocytes to intestinal lymphoid tissues and is critical for the homing of mucosal plasmablasts to the gut [17]. Therefore, the presence of circulating poliovirus-specific ASCs expressing α4β7 indicates recent or ongoing intestinal exposure to poliovirus. Exposure to live poliovirus, through OPV immunization, has been shown to induce an increase in poliovirus-specific ASCs expressing α4β7 integrin [5, 18]. However, the relationship between virus-specific ASCs expressing α4β7 integrin and mucosal protection against poliovirus is unknown.

The primary objective of this study was to explore the potential value of poliovirus-specific blood ASCs, including gut-derived α4β7^+^ ASCs, as proxy markers of mucosal immune protection against poliovirus excretion. A secondary objective was to explore the relationship between such ASCs and seroprotection (i.e. antibody-mediated virus neutralization) after poliovirus vaccination. In this paper, we expand on the results of a trial previously published [19] exploring the boosting effects of inactivated poliovirus vaccine (IPV) (for serotypes 1, 2 and 3) and bivalent 1 and 3 oral poliovirus vaccine (bOPV) in children previously immunized with OPV. For these purposes, a micro-modified ELISPOT assay was developed to allow point-of-site detection of ASC responses, including gut-homing α4β7-expressing plasma blasts, in small volumes of whole (unfractionnated) blood [9] under field settings.

## Materials and Methods

### Study Design and Methods

The study was approved by the Drugs Controller General (India), the Institutional Review Boards of the World Health Organization (WHO), the US Centers for Disease Control and Prevention (CDC), and the International Vaccine Institute (IVI). The study was registered at Clinical Trial Registry of India (Reference: CTRI/2011/09/002018).

This study was nested within a large clinical trial carried out in children residing in Moradabad District, Uttar Pradesh State, India [19] to assess the efficacy of IPV in boosting mucosal immunity. In the clinical trial, subjects received IPV, bOPV or no vaccine. A bOPV challenge was administered four weeks later and excretion was assessed 3, 7 and 14 days later. The overall study included children from three age groups (6–11 months, 5 years or 10 years). Complete methods of the clinical trial describing overall study design, participant inclusion and sample collection were described previously [19].

Of the 330 children in the 10 years age group enrolled in the main clinical trial, 200 were planned to be enrolled for the ELISPOT test. Recruitment of required numbers was done from all 10 study sites over last four days, limiting the participation per day on first come first serve basis. Finally 199 children were enrolled in the study. Participants were recruited from 13–16 October 2011 and only one follow up 7 days after i.e. 20–23 October 2011.Written informed consent from the parent and assent from the participating child was taken for study participation including the additional blood sample of 3 ml at days 0 and 7 of the study. Informed consent form (ICF) and the Assent forms were approved by the applicable Ethics Committees and IRBs.

### Laboratory Analyses

In the main study, blood specimens (3 ml) were collected by venipuncture at baseline (i.e. day 0) and at days 28 and 56 after intervention (either IPV, bOPV or no vaccination). Serum samples were tested for neutralizing antibody titers to poliovirus types 1, 2, and 3, using a micro-neutralization assay[20]. Seroconversion was defined as a change from a negative (baseline titer <1:8) to a positive (≥1:8) titer after vaccine administration [20–22] and boosting as a 4-fold rise in antibody titer for children with a baseline titer of 1:8–1:362 [23]. Stool specimens were collected on day 28 (before challenge) and 3, 7 and 14 days (i.e. on day 31, 35 and 42) after challenge with OPV, and titrated for poliovirus types 1, 2 and 3 as per WHO guidelines[3]. Data are expressed as log10 CCID50/gram of feces.

Measurements of blood ASC responses from all samples were performed at two field laboratory stations on 3 ml samples of anti-coagulated blood collected before (day 0) and one week (day 7) after booster intervention (immunization with IPV or bOPV), or control (no vaccination). All samples were stored in cooled (5–10°C) containers and tested within 4 hours after collection. A micro-modified version of the original ELISPOT assay was adapted for simultaneous detection of magnetically enriched blood ASCs secreting IgA or IgG to poliovirus 1, 2 and 3 (inactivated whole virus) [9]. Briefly, EDTA-treated blood was mixed with a red blood cell lysis solution for 5 min, washed with PBS-EDTA buffer by centrifugation, and re-suspended to the initial blood volume (3 ml) with PBS-EDTA buffer. Human ASCs were enriched from lysed blood using a mixture (1:1) of magnetic beads coated with monoclonal antibodies to HLA-DR and CD19, followed by application of a magnetic field [15]. Although a variety of monoclonal antibodies specifying cell surface markers that are selectively expressed by human B cells or plasmablasts (but not by resting T cells and granulocytes, which are abundant in blood), such as CD19, HLA-DR, sIg, CD27 or CD138, can be used for this purpose. But in our experience the combination of both of HLA-Dr and CD19 coated beads is most effective for enriching human blood ISCs and ASCs, resulting in near-complete reduction (90–95%) of FACS-detectable cells in the negative fraction [9]. Further this enrichment steps avoids Ficoll gradient purification, which needs large blood volume and results in higher background due to RBC contamination and lower sensitivity of the ELISPOT assay. Our current approach of enrichment of ASCs overcome the limitation of small volume of blood available for immunological studies, particularly in infants and children. For 1 ml of blood sample 25 μl each of HLA-Dr and CD19 coated beads were used. The latter ASCs are being referred to as nominal “total” ASCs. Nominal “mucosal ASCs” were isolated from 1.0 ml lysed blood samples using magnetic beads coated with monoclonal antibodies to α4β7. After magnetic capture, free and cell-bound beads were washed and re-suspended to the original blood volume in serum‐free medium prior to being assayed for ASC numbers. These separation procedures routinely yield negative fractions depleted by more than 95% of FACS-detectable HLA-DR^+^, CD19^+^ cells and by more than 90% α4β7^+^ cells [24], respectively. Moreover, negatively sorted cells contain less than 1% ELISPOT detectable immunoglobulin-secreting cells (ISCs,.

For antigen-specific ASC enumerations, ELISPOT wells were coated with purified killed poliovirus type 1, 2, or 3 and control antigen (i.e. Bovine Serum Albumin, BSA) as described elsewhere [9]. Similarly, immunoglobulin-secreting cells (ISCs) irrespective of antigen specificity were enumerated in parallel wells coated with a mixture of affinity-purified goat antibodies to human Ig k and λ light chains. All coated plates were dried and kept in individual sealed aluminium bags with a desiccator prior to being used (within 3 months). After incubation of ASC- and ISC-containing cell suspensions for 3 hours at 37°C in a battery powered portable incubator (Milipore^®^), wells were extensively washed with PBS-EDTA and PBS-Tween 20. Next, a mixture of appropriately diluted goat antibodies to human IgA and IgG, respectively labeled with alkaline phosphatase and horseradish peroxidase, was added to the wells and incubated for one hour. After washings, zones of solid phase-bound secreted IgA and IgG antibodies were visualized by stepwise incubation with corresponding enzyme chromogen substrates followed by washing with water [9]. After drying, plates were scanned and blue (IgA) and red (IgG) spots enumerated using an automated ELISPOT reader.

Total and mucosal ASCs as well as ISCs were enumerated against each of poliovirus type 1, 2 and 3 antigens and net ASC and ISC counts were determined after subtracting corresponding non-specific counts detected in control (BSA-coated) wells. Data are expressed as ASC or ISC numbers per milliliter of blood.

### Data Analyses

Numbers of poliovirus-specific IgA-and IgG-producing blood ASCs to each of the three serotypes of poliovirus were determined at day 0 and day 7. ASC responses were further differentiated into total (HLA-DR^+^/CD19^+^) and mucosal (α4β7^+^) ASCs. ELIPSOT data for the three arms (no vaccine, bOPV, IPV) were combined when compared to excretion and seroneutralization data.

The primary outcome measure of the study was a low mucosal gut-homing (i.e. α4β7+) IgA- and/or IgG-ASC response in subjects excreting virus following challenge with bOPV. Excretion was defined as any excretion 3, 7 or 14 days after challenge (i.e. on day 31, 35 or 42). The secondary outcome measure was an increased total IgA- and/or IgG-ASC response in subjects with a humoral immune response, defined as either a change from seronegative to seropositive (i.e., a reciprocal titer ≥ 8) [20–22], or a four-fold or higher increase in antibody titer [23].

Median mucosal (α4β7^+^) and total ASC counts were compared to virus excretion and serological status, respectively, using the non-parametric Wilcoxon rank-sum test. The median 95% confidence intervals were calculated using bootstrapping with 10,000 replications. Subjects were further classified as mucosal and total ASC responders when his/her mucosal or total IgA- or IgG-ASC count 7 days post-vaccination was ≥ three-fold higher than the mean baseline corresponding ASC count (i.e. day 0) plus one standard deviation [9]. Chi-squared tests were used to compare the prevalence of total and α4β7^+^ ASC responses according to immune response status and poliovirus excretion. Sensitivity (Se), Specificity (Sp), Positive (PPV) and Negative Predictive Values (NPV) were calculated to assess the predictive ability of ASC testings when compared to viral shedding and serum neutralizing antibodies, respectively. P-values <0.05 were considered significant. Statistical analyses were performed in R 3.1.2 [25].

## Results

**Fig 1:**
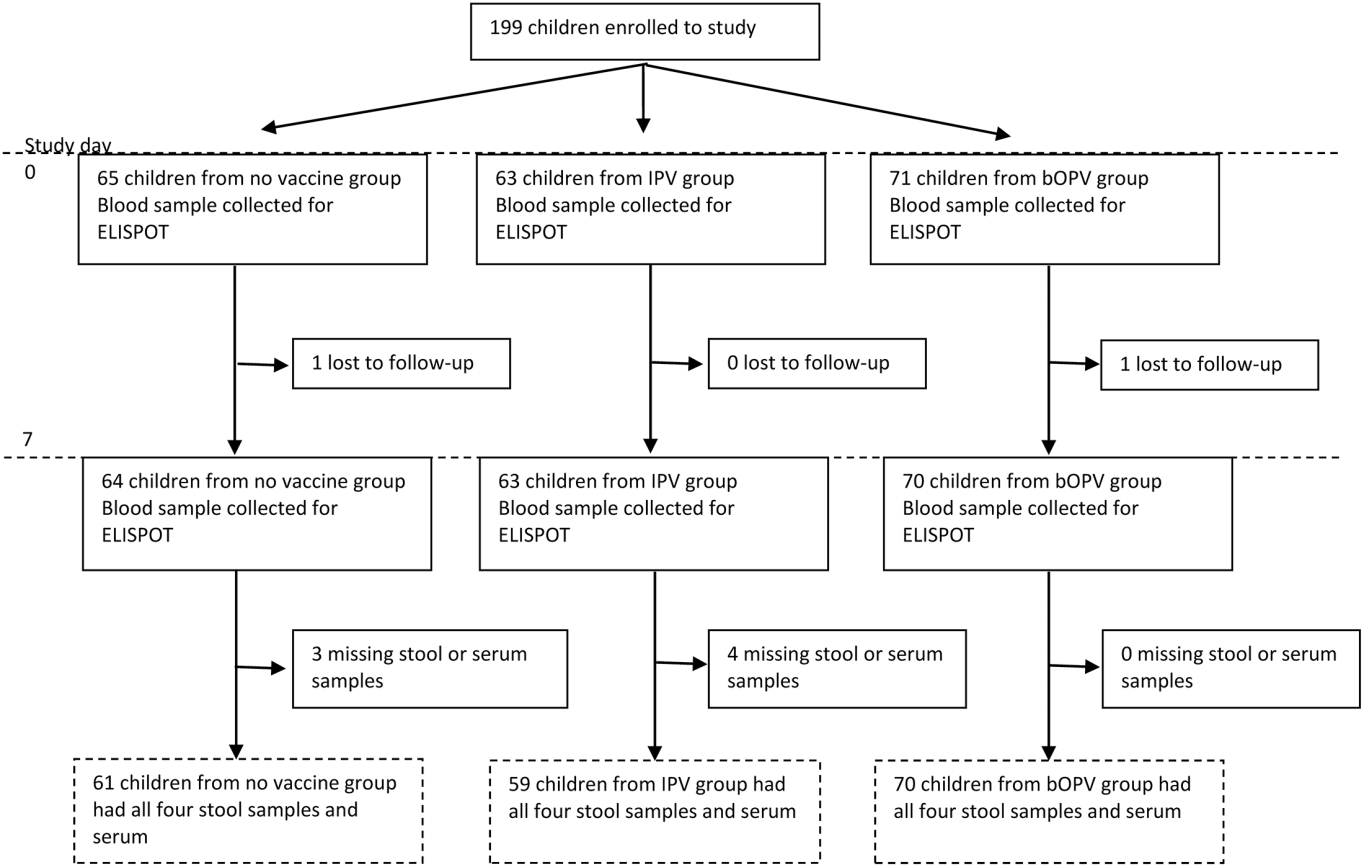
CONSORT flow diagram of included subjects. bOPV = bivalent Oral Polio Vaccine; IPV = Inactivated Polio Vaccine; ELISPOT = Enzyme-Linked ImmunoSpot.

Of the 199 subjects enrolled in this study, 190 (95.5%) had complete data and were included in the analysis. Subjects were excluded if they were lost to follow up or if their blood sample was rejected due to clotting or hemolysis during transport to field laboratories (Fig 1). Of the 190 subjects, one was excreting poliovirus type 1 and seven excreting type 3 at day of bOPV challenge. All subjects excreting virus before challenge were in the bOPV arm. These subjects were included in the analysis; however, exclusion of these subjects produced similar results.

### A single IPV boost induces mucosal ASC responses in blood

High numbers of IgA- and IgG-ASCs, including α4β7^+^ ASCs, were detected in blood samples from IPV-vaccinated individuals 7 days after vaccination (Table 1). The IPV boost induced α4β7^+^ IgA- and IgG- ASC responses of higher magnitude as compared to an OPV boost. Moreover, blood mucosal α4β7^+^ IgA- and IgG-ASC counts were highest to poliovirus type 2 in the IPV group. Total ASC responses were also of higher magnitude in the IPV group compared to the OPV group.

**Table 1:** Median mucosal α4β7+ and total IgA and IgG ASCs at day 7 by intervention group and poliovirus serotype.

				Poliovirus Serotype 1		Poliovirus Serotype 2		Poliovirus Serotype 3	
	ASC	Vaccine type	N	Median (95% CI)	P-value	Median (95% CI)	P-value	Median (95% CI)	P-value
IgA	α4β7^+^	No Vaccine	61	0.0 (0.0–0.0)	Ref	0.0 (0.0–0.0)	Ref	0.0 (0.0–0.0)	Ref
		IPV	59	5660.4 (3597.1–12552.3)	<0.001	31380.8 (25252.5–41294.6)	<0.001	10162.6 (5649.7–15957.5)	<0.001
		bOPV	70	0.0 (0.0–0.0)	0.262	0.0 (0.0–0.0)	0.918	0.0 (0.0–0.0)	0.021
	Total	No Vaccine	61	0.0 (0.0–666.7)	Ref	0.0 (0.0–0.0)	Ref	0.0 (0.0–0.0)	Ref
		IPV	59	21317.8 (12872.6–33475.8)	<0.001	48728.8 (36072.1–72916.7)	<0.001	20325.2 (14166.7–33333.3)	<0.001
		bOPV	70	0.0 (0.0–2224.0)	0.344	0.0 (0.0–0.0)	0.874	0.0 (0.0–2608.7)	0.007
IgG	α4β7^+^	No Vaccine	61	0.0 (0.0–0.0)	Ref	0.0 (0.0–0.0)	Ref	0.0 (0.0–0.0)	Ref
		IPV	59	7668.7 (4189.9–13888.9)	<0.001	11363.6 (7462.7–14534.9)	<0.001	2688.2 (1728.1–3846.2)	<0.001
		bOPV	70	0.0 (0.0–0.0)	0.114	0.0 (0.0–0.0)	0.100	0.0 (0.0–0.0)	0.062
	Total	No Vaccine	61	0.0 (0.0–3731.3)	Ref	0.0 (0.0–0.0)	Ref	0.0 (0.0–0.0)	Ref
		IPV	59	25914.6 (18518.5–38888.9)	<0.001	29411.8 (25000–37650.6)	<0.001	10000.0 (6329.1–13652.6)	<0.001
		bOPV	70	1516.0 (0.0–4374.1)	0.431	0.0 (0.0–0.0)	0.307	0.0 (0.0–776.4)	0.472

CI: confidence interval; N = total number of subjects; Ref = Reference; IPV = Inactivated Polio Vaccine; bOPV = bivalent Oral Polio Vaccine; P-value calculated using Wilcoxon rank-sum test. 95% CIs calculated using bootstrapping with 10,000 replications.

Median total IgA-ASC numbers/ ml of blood to poliovirus type 1, 2 and 3 after an IPV booster injection were 21317.8 (95% CI: 12872.6–33475.8), 48728.8 (36072.1–72916.7) and 20325.2 (14166.7–33333.3),, respectively. A large proportion of virus-specific total IgA-ASCs was accounted for by α4β7^+^ ASCs, ranging from approximately 27% for poliovirus type 1 (median α4β7^+^ IgA-ASCs/ml: 5660.4 (95% CI: 3597.1–12552.3)), to 64% for poliovirus type 2 (median α4β7^+^ IgA-ASCs/ml: 31380.8 (25252.5–41294.6)), and 50% of total IgA-ASCs for poliovirus type 3 (median α4β7^+^ IgA-ASCs/ml: 10162.6 (5649.7–15957.5)) (Table 1). In the OPV group, median total IgA-ASCs numbers were substantially lower with values of 0.0 (0.0–2224.0), 0.0 (0.0–0.0) and 0.0 (0.0–2608.7) for poliovirus type 1, 2 and 3, respectively. Similar results were found for total as well as α4β7^+^ IgG ASCs to all poliovirus serotypes (Table 1). Thus, a single IPV booster dose elicited substantially higher IgA- and IgG-ASC responses, including higher frequencies of α4β7^+^ IgA- and IgG-ASCs, than a booster dose of OPV in children previously immunized with OPV.

**Fig 2:**
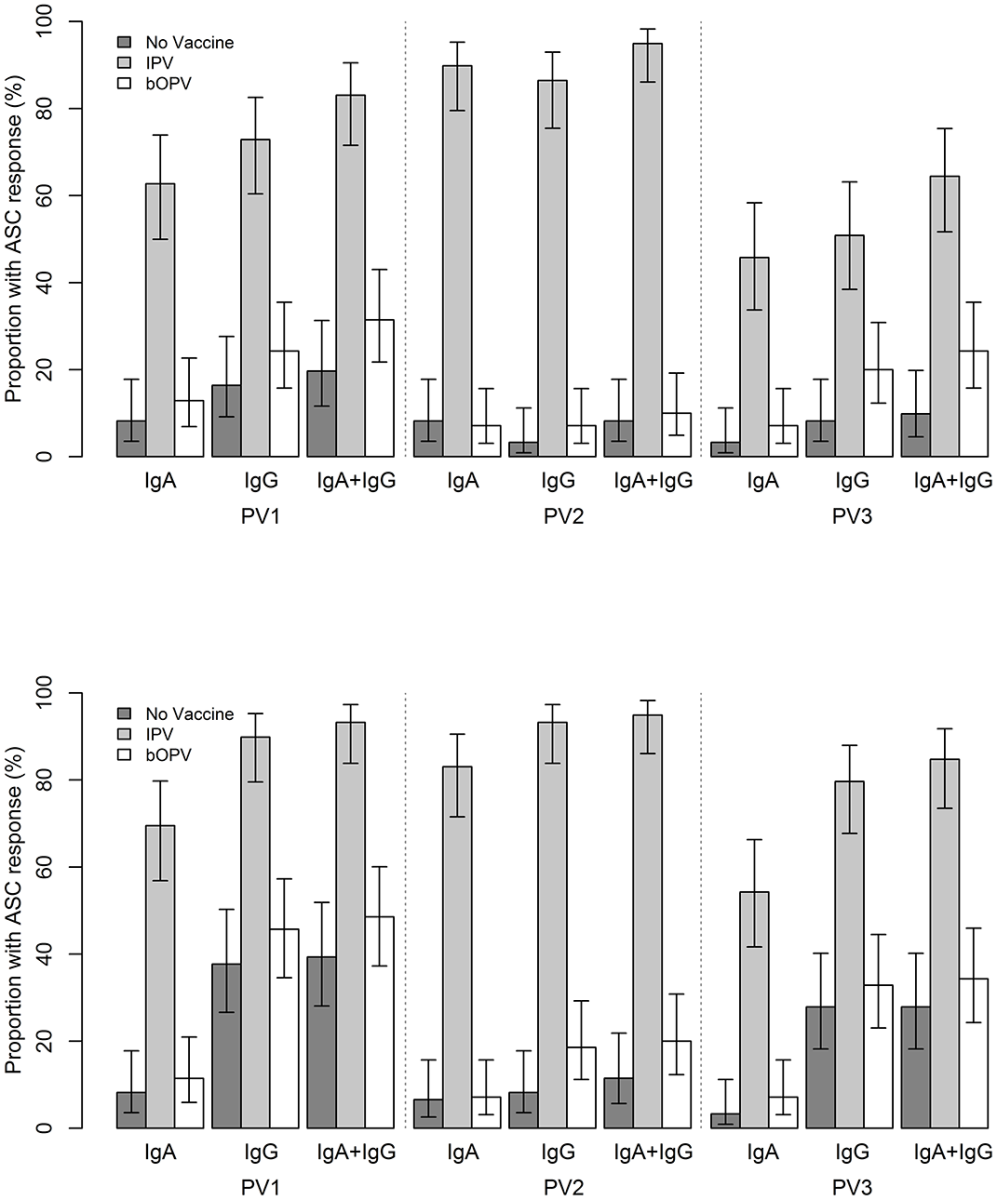
**a:** Proportion of subjects with α4β7+ ASC IgA and/or IgG response by intervention group and poliovirus serotype. **b:** Proportion of subjects with total ASC IgA and/or IgG response by intervention group and poliovirus serotype.

The proportion of subjects classified as IgA- and/or IgG-ASC responders was also highest in the IPV group, irrespective of poliovirus serotype (Fig 2a and 2b). Overall, for poliovirus types 1, 2 and 3, the proportion of subjects with a positive α4β7^+^ IgA-ASC response was 26.8% (95% CI: 21.0–33.6), 33.2% (26.9–40.1) and 17.9% (13.1–24.0), for poliovirus serotype 1, 2 and 3 respectively, and the proportion of subjects with a positive α4β7+ IgG-ASC response was 36.8% (30.3–43.9), 30.5% (24.4–37.4) and 25.8% (20.1–32.4), respectively. Likewise, a similar tendency was observed for total ASCs to poliovirus type 1, 2 and 3, with proportions of responder subjects of 28.4% (95% CI: 22.5–35.2), 30.5% (24.4–37.4) and 20.5% (15.4–26.8) for total IgA-ASCs to poliovirus type 1, 2, and 3, respectively, and of 56.8% (49.7–63.7), 38.4% (31.8–45.5) and 45.8% (38.9–52.9) for corresponding poliovirus type-specific total IgG-ASCs.

### Excretion and immune response by intervention group

**Fig 3:**
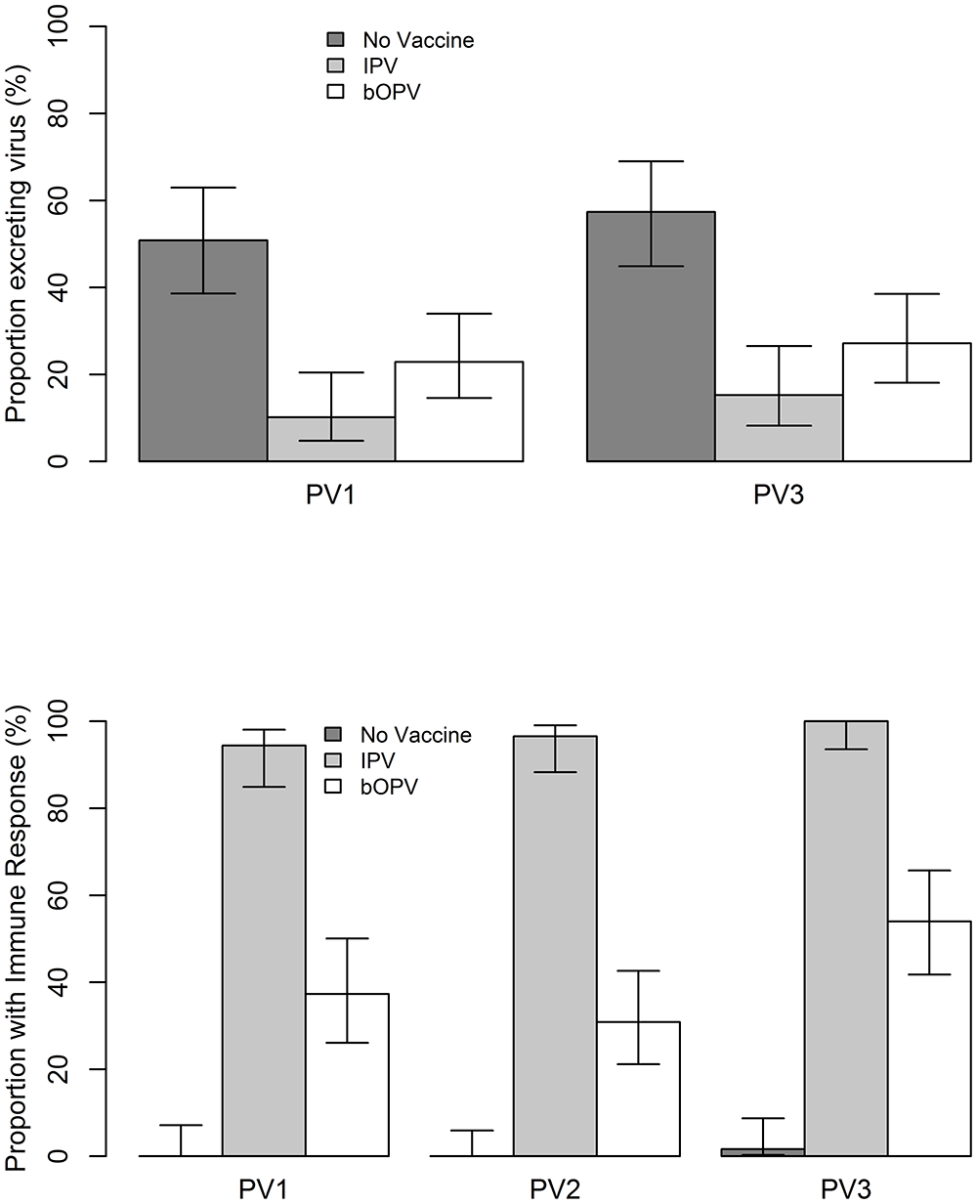
**a:** Proportion of subjects excreting virus after booster vaccination with OPV and IPV vaccines by intervention group and poliovirus serotype. PV1: Poliovirus type 1; PV2: Poliovirus type 2; PV3: Poliovirus type 3; IPV: Inactivated Polio Vaccine; bOPV = bivalent Oral Polio Vaccine. **b:** Proportion of subjects with immune response after booster vaccination with OPV and IPV vaccines by intervention group and poliovirus serotype.

Any fecal shedding of poliovirus types 1 and 3 was 10.2 (95% CI: 4.7–20.5) and 15.3 (8.2–26.5), respectively, in the IPV group, 22.9 (14.6–34.0) and 27.1 (18.1–38.5) in the bOPV group and 50.8 (38.6–62.9) and 57.4 (44.9–69.0) in the control group (Fig 3a). An immune response to poliovirus type 1 was demonstrated in 94.4 (84.9–98.1), 37.3 (26.1–50.1) and 0.0 (0.0–7.1) of subjects in the IPV, bOPV and no vaccine groups, respectively (Fig 3b). Similar results were found for serotypes 2 (96.6 (88.3–99.1), 30.9 (21.2–42.6) and 0.0 (0.0–5.9)) and 3 (100.0 (93.6–100.0), 54.0 (41.8–65.7) and 1.6 (0.3–8.7)).

### Correlation between blood ASC responses and poliovirus excretion

The proportion of subjects ever excreting poliovirus for type 1 and 3 after challenge with bOPV was 27.9% (95% CI: 22.0–34.7) and 33.2% (26.9–40.1), respectively.

The median number of α4β7+ IgA-ASCs in subjects excreting poliovirus type 3 was significantly lower compared to subjects not excreting virus (p<0.001). Similar results were found for polio type 3-specific α4β7^+^ IgG-ASC responses (p = 0.029). This relationship was less pronounced when considering excretion of poliovirus type 1 and α4β7^+^ IgA- (p = 0.190) and IgG- (p = 0.067) ASCs.

Classifying subjects as α4β7+ ASC responders or non-responders disclosed a significant association between the proportion of α4β7^+^ IgA-ASC and/or IgG-ASC responders and absence of excretion for poliovirus type 3 (IgA: p = 0.015; IgG: p = 0.078; IgA**+**IgG: p = 0.021) (Table 2). For poliovirus type 1, a similar trend appeared; however, it was not statistically significant (IgA p = 0.469; IgG: p = 0.314; IgA+IgG: p = 0.195).

**Table 2:** Median mucosal α4β7+ ASCs at day 7 and proportion of subjects with mucosal α4β7^+^ IgA and/or IgG ASC response by excretion status of poliovirus serotypes 1 and 3.

Poliovirus Serotype	α4β7^+^ASC	Excretion status	N	Median (95% CI)	P-value	n	% ASC Response (95% CI)	P-value
Serotype 1	IgA	No Excretion	137	0.0 (0.0–1420.5)	0.091	39	28.5 (21.6–36.5)	0.469
		Excretion	53	0.0 (0.0–0.0)		12	22.6 (13.5–35.5)	
	IgG	No Excretion	137	0.0 (0.0–1728.1)	0.128	54	39.4 (31.6–47.8)	0.314
		Excretion	53	0.0 (0.0–0.0)		16	30.2 (19.5–43.5)	
	IgA + IgG	No Excretion	137	NA	NA	64	46.7 (38.6–55.1)	0.195
		Excretion	53	NA		19	35.9 (24.3–49.3)	
Serotype 3	IgA	No Excretion	127	1225.5 (0.0–2500.0)	<0.001	29	22.8 (16.4–30.9)	0.015
		Excretion	63	0.0 (0.0–0.0)		5	7.9 (3.4–17.3)	
	IgG	No Excretion	127	0.0 (0.0–844.6)	0.007	38	29.9 (22.6–38.4)	0.078
		Excretion	63	0.0 (0.0–0.0)		11	17.5 (10.0–28.6)	
	IgA + IgG	No Excretion	127	NA	NA	48	37.8 (29.8–46.5)	0.021
		Excretion	63	NA		13	20.6 (12.5–32.2)	

CI: confidence interval; N = total number of subjects; n = number of subjects with an ASC response; % = proportion; IgA + IgG = either IgA- or IgG- α4β7+ASCs; Excretion = any excretion following bOPV challenge (i.e. day 31, 35 or 42). P-values for ASC counts and proportion with ASC response calculated using Wilcoxon rank-sum test and Chi-squared test, respectively. Median 95% CIs calculated using bootstrapping with 10,000 replications.

Using excretion as gold standard, we assessed the Se, Sp, PPV and NPV of α4β7+ IgA- and/or IgG-ASCs (Table 3). For poliovirus type 1, the Se and Sp when considering IgA- and IgG-ASCs was 47% (95% CI: 38–55) and 64% (50–77), with PPV and NPV of 77% (67–86) and 32% (23–41), respectively. For poliovirus type 3, the Se and Sp when considering IgA and IgG ASCs was 38% (95% CI: 29–47) and 79% (67–89), with PPV and NPV of 79% (66–88) and 39% (30–48), respectively.

**Table 3:** Sensitivity, specificity, positive and negative predictive values of mucosal α4β7^+^IgA- and/or IgG- ASC responses.

		α4β7+ ASC				Total ASC			
		Sensitivity	Specificity	PPV	NPV	Sensitivity	Specificity	PPV	NPV
Poliovirus Serotype	α4β7^+^ASC	% (95% CI)	% (95% CI)	% (95% CI)	% (95% CI)	% (95% CI)	% (95% CI)	% (95% CI)	% (95% CI)
Serotype 1	IgA	28 (21–37)	77 (64–88)	76 (63–87)	29 (22–38)	55 (43–66)	88 (79–94)	78 (65–89)	71 (61–79)
	IgG	39 (31–48)	70 (56–82)	77 (66–86)	31 (23–40)	73 (61–82)	56 (45–66)	57 (46–67)	71 (59–82)
	IgA + IgG	47 (38–55)	64 (50–77)	77 (67–86)	32 (23–41)	75 (64–85)	53 (43–64)	57 (46–67)	73 (60–83)
Serotype 2	IgA	NA	NA	NA	NA	62 (51–73)	92 (85–96)	84 (72–93)	78 (70–85)
	IgG	NA	NA	NA	NA	71 (60–81)	85 (76–91)	76 (65–86)	81 (72–88)
	IgA + IgG	NA	NA	NA	NA	74 (63–83)	83 (74–89)	75 (64–84)	82 (74–89)
Serotype 3	IgA	23 (16–31)	92 (82–97)	85 (69–95)	37 (30–45)	36 (26–47)	94 (87–98)	87 (72–96)	59 (51–67)
	IgG	30 (22–39)	83 (71–91)	78 (63–88)	37 (29–45)	65 (54–75)	73 (63–82)	71 (60–81)	67 (57–76)
	IgA + IgG	38 (29–47)	79 (67–89)	79 (66–88)	39 (30–48)	68 (58–78)	72 (61–81)	71 (61–80)	69 (58–78)

CI: confidence interval; IgA + IgG: either IgA- or IgG- α4β7+ ASC response; PPV: positive predictive value; NPV: negative predictive value.

### Correlations between poliovirus-specific blood ASCs and sero-protection

The proportion of subjects with a systemic immune response following vaccination (i.e., seroconversion or four-fold increase in serum neutralizing antibody titer) against types 1, 2 and 3 were 44.8% (95% CI 37.4–52.5), 41.2% (34.4–48.3) and 50.6% (43.3–57.8), respectively.

The median numbers of IgA- and IgG-ASCs were significantly greater in subjects with a serum neutralizing antibody response to poliovirus compared to those without, irrespective of serotype (P<0.001). Similar results were found when classifying subjects as IgA and/or IgG ASC responders (Table 4).

**Table 4:** Proportion of subjects with IgA- and/or IgG-ASC responses to poliovirus types 1, 2 and 3 and systemic immune response (seroconversion or 4-fold rise in neutralizing antibody titers).

Poliovirus Serotype	Total ASC	Immune Response (IR) Status	N	Median (95% CI)	P-value	n	% ASC Response (95% CI)	P-value
Serotype 1	IgA	No IR	90	0.0 (0.0–0.0)	<0.001	11	12.2 (7.0–20.6)	<0.001
		IR	73	12872.6 (6493.5–25380.7)		40	54.8 (43.4–65.7)	
	IgG	No IR	90	0.0 (0.0–0.0)	<0.001	40	44.4 (34.6–54.7)	<0.001
		IR	73	15923.6 (9868.4–25547.4)		53	72.6 (61.4–81.5)	
	IgA + IgG	No IR	90	NA	NA	42	46.7 (36.7–56.9)	<0.001
		IR	73	NA		55	75.3 (64.4–83.8)	
Serotype 2	IgA	No IR	110	0.0 (0.0–0.0)	<0.001	9	8.2 (4.4–14.8)	<0.001
		IR	77	32092.4 (14634.2–45348.8)		48	62.3 (51.2–72.3)	
	IgG	No IR	110	0.0 (0.0–0.0)	<0.001	17	15.5 (9.9–23.4)	<0.001
		IR	77	24779.7 (18115.9–32894.7)		55	71.4 (60.5–80.3)	
	IgA + IgG	No IR	110	NA	NA	19	17.3 (11.4–25.4)	<0.001
		IR	77	NA		57	74.0 (63.3–82.5)	
Serotype 3	IgA	No IR	89	0.0 (0.0–0.0)	<0.001	5	5.6 (2.4–12.5)	<0.001
		IR	91	11111.1 (5814.0–16736.4)		33	36.3 (27.1–46.5)	
	IgG	No IR	89	0.0 (0.0–0.0)	<0.001	24	27.0 (18.8–37.0)	<0.001
		IR	91	7299.3 (4310.3–10000.0)		59	64.8 (54.6–73.9)	
	IgA + IgG	No IR	89	NA	NA	25	28.1 (19.8–38.2)	<0.001
		IR	91	NA		62	68.1 (58.0–76.8)	

ASC = antigen secreting cells; IR = immune response; CI: confidence interval; N = total number of subjects with potential for immune response; n = number of subjects with an ASC response; % = proportion; IgA + IgG = either IgA- or IgG- α4β7+ASCs; P-values for ASC counts and proportion with ASC response calculated using Wilcoxon rank-sum test and Chi-squared test, respectively. Median 95% CIs calculated using bootstrapping with 10,000 replications.

Using systemic immune response) as reference, we assessed the Se, Sp, PPV and NPV of IgA- and IgG-ASC testings. For poliovirus type 1, the Se and Sp when considering IgA- and IgG-ASCs were 75% (95% CI: 64–85) and 53% (43–64), with PPV and NPV of 57% (46–67) and 73% (60–83), respectively. For poliovirus type 2, the Se and Sp when considering IgA- and IgG-ASCs were 74% (95% CI: 63–83) and 83% (74–89), with PPV and NPV of 75% (64–84) and 82% (74–89), respectively. For poliovirus type 3, the Se and Sp when considering IgA- and IgG-ASCs were 68% (95% CI: 58–78) and 72% (61–81), with PPV and NPV of 71% (61–80) and 69% (58–78), respectively.

## Discussion

In the present study, we measured blood ASCs to poliovirus, including gut homing α4β7+ ASCs, after intervention with a single booster vaccination with OPV or IPV and in controls (no vaccination) in Indian children with prior history of OPV immunization. In both the OPV and IPV arms, α4β7^+^ IgA- and IgG-ASC responses were observed after one vaccine dose, suggesting preferential mucosal homing of α4β7^+^ ASCs from and to the gut. However, in the OPV arm the proportion of subjects responding on day 7 was significantly lower than in the IPV arm and the magnitude of their ASC responses was substantially lower. The significantly higher mucosal ASC responses observed after parenteral administration of a single dose of IPV is consistent with the lower proportion of children excreting virus in the IPV group when compared to the OPV group observed in this trial [19] and in another recent report in younger children from southern India [26]. This observation suggests that mucosal memory B cells, as opposed to their differentiated plasma cell progenitors, may be more broadly distributed and can be mobilized from extra-mucosal tissues draining the site of vaccine injection. Alternatively, antigen-presenting cells in peripheral tissues draining the site of IPV injection may be endowed more tissue promiscuous migratory properties and more efficient stimulatory properties to activate memory cells in mucosal tissues.

An unexpected finding was the remarkably high α4β7^+^ ASC responses seen in subjects receiving an IPV booster dose although most children living in the area studied are expected to have been previously exposed to and/or immunized with monovalent (serotype 1) or bivalent (serotypes 1 and 3) OPV, and tOPV. While shared, including cross-neutralizing, epitopes have been described on poliovirus type 1 and 2 [27, 28]. The latter observation suggests that mucosal ASC responses are broadly cross-reactive and that mucosal immunological memory to such cross-reactive poliovirus epitopes is of longer duration than expected.

A significant association was found between α4β7^+^ ASC responses and lack of excretion of poliovirus type 3. However, only a weak association was observed for poliovirus type 1. The lack of significant association for type 1 could be due to higher exposure to type 1 antigen in these children through previous vaccination campaigns with monovalent OPV1 in the Moradabad region, consistent with the high seroprevalence of this serotype in this region [29, 30]. In these children, pre-existing mucosal antibodies to type 1 poliovirus may have inhibited replication of OPV-derived poliovirus type 1 in intestinal and/or pharyngeal tissues after challenge, irrespective of whether or not an ASC response had been induced following booster vaccination.

The ability of blood α4β7^+^ IgA and/or IgG ASCs to serve as proxy markers of mucosal immune protection against poliovirus to was high (i.e. high PPV) for both type 1 and type 3 when considering excretion as gold standard; therefore, an α4β7^+^ IgA- and/or IgG-ASC response was highly indicative of a concurrent mucosal immune response. However, a large proportion of subjects with reduced or no virus excretion failed to mount a detectable α4β7+ IgA- and/or IgG-ASC response and as a result went undetected (i.e. low NPV). This could be due, in part to the time frame (7 days post vaccination) selected for blood collection which may have been suboptimal. Exploring a range of time points of blood collection could help improving the predictive ability of this marker. Blood IgA- and IgG-ASC responses, irrespective of α4β7 expression, were significantly greater in subjects with a systemic neutralizing antibody response to all 3 poliovirus serotypes compared to those without (P<0.001). The predictive ability of such ASCs to correctly identify subjects with seroprotection was relatively high for all serotypes. However, such ASC testing does not indicate a functional property of secreted antibodies and is thus unlikely to replace virus-neutralization assays as surrogate marker of protective humoral immunity to poliovirus.

In the present trial the potential value of poliovirus-specific blood ASCs, and especially α4β7^+^ ASCs, as biomarker of mucosal immunity has been documented. However, important study limitations must be mentioned. As this was an exploratory study, the children included in the study were not naive and had previously been exposed to repeated OPV doses and/or circulating wild-type poliovirus (mainly type 1) prior to this trial. Because prior exposure to poliovirus type 1 may have interfered with OPV1 replication in the mucosa, hiding a possible association between ASC responses and fecal virus excretion, the findings for poliovirus type 3 are likely more representative of the true relationship between circulating mucosal ASCs and virus excretion. Repeating the study in a population of naïve children, i.e. newborns and young infants, could shed light on this issue. Furthermore, exploring a range of time points for blood collection for ELISPOT analyses could help validate such an association as some children may respond earlier than 7 days. On the other hand, the program is likely most interested in assessing mucosal immunity after a complete series with 3–4 doses of OPV vaccines.

This study has explored the potential value of virus-specific blood ASCs, including α4β7+ ASCs, as a surrogate marker of polio vaccine-induced mucosal immune protection. This study also indicates that blood ASCs provide an early marker of the systemic immunogenicity of these vaccines and are predictive of virus neutralizing antibody responses that peak later in serum. However, at that stage and at variance with poliovirus type 3, such blood ASC responses do not provide a sufficiently reliable surrogate of mucosal immune protection to poliovirus type 1. Further studies in subjects with and without history of prior exposure to poliovirus vaccines are needed to validate or not the value of such ASCs as surrogates of vaccine-induced mucosal protection.

## Supporting Information

S1 File Study Protocol. (DOC)

S2 File Study Data. (XLS)
