# Supplementary material for: Human Circulating Antibody-Producing B Cell as a Predictive Measure of Mucosal Immunity to Poliovirus
Source: PLoS One. 2016 Jan 5;11(1):e0146010. doi: 10.1371/journal.pone.0146010 (PMC4701219; doi:10.1371/journal.pone.0146010)
Supplement: S1 File — (DOC) [file pone.0146010.s001.doc]

STUDY PROTOCOL

***Protocol title****:*

**ASSESSMENT OF MUCOSAL IMMUNITY TO POLIOVIRUSES AFTER A SUPPLEMENTAL DOSE OF BIVALENT ORAL POLIOVIRUS VACCINE OR INACTIVATED POLIOVIRUS VACCINE IN CHILDREN IN NORTHERN INDIA: A RANDOMIZED TRIAL**

**Protocol Number: PBL/CR/2011/02/CT**

**Version:** 08 of 13 July 2011

SPONSOR: Panacea Biotec Limited

### **PRINCIPAL INVESTIGATOR:** Dr. Jagadish M. Deshpande

Enterovirus Research Centre (ERC), Indian Council for Medical Research (ICMR), Mumbai, India

**CO- Investigator:** Dr Hamid Jafari

Project Manager, National Polio Surveillance Project (NPSP)

GOI and WHO Collaboration, New Delhi, India

STUDY INSTITUTIONS:

Indian Council of Medical Research (ICMR), Delhi, India

National Polio Surveillance Project (NPSP), Delhi, India

Ministry of Health and Family Welfare, Government of India , Delhi, India

Department of Health and Family Welfare, Government of Uttar Pradesh, Lucknow, India

World Health Organization (WHO), Geneva, Switzerland

Centers for Disease Control and Prevention (CDC), Atlanta, USA

The International Vaccine Institute (IVI), Korea

Imperial College, London

Amendment details:

| **Amendment No.** | **Date** | **Authorized Signatory** |
| --- | --- | --- |
|  |  |  |

CONFIDENTIALITY STATEMENT

The information contained herein is the property of Panacea Biotec Ltd. and cannot be reproduced, published or disclosed to others without written authorization of Panacea Biotec Ltd.

**PROTOCOL APPROVAL SHEET**

# Panacea Biotec Ltd.

**ASSESSMENT OF MUCOSAL IMMUNITY TO POLIOVIRUSES AFTER A SUPPLEMENTAL DOSE OF BIVALENT ORAL POLIOVIRUS VACCINE OR INACTIVATED POLIOVIRUS VACCINE IN CHILDREN IN NORTHERN INDIA: A RANDOMIZED TRIAL**

**PROTOCOL NO.: PBL/CR/2011/02/CT, Version dated**

PROTOCOL PREPARED BY: Panacea Biotec Ltd.

PROTOCOL APPROVED BY:

___________________________ ________________________

(Dr. Arani Chatterjee) Date

VP-Clinical Research

Panacea Biotec Ltd.

**LIST OF ABBREVIATIONS**

- ACPE The Advisory Committee for Polio Eradication
- AE Adverse Event
- bOPV Bivalent Oral Polio Vaccine
- CI Confidence Interval
- CRA Clinical Research Associate
- CRFs Case Report Forms
- CDC Centers for Disease Control and Prevention
- DCG(I) Drug Controller General of India
- DSMB Data Safety Monitoring Board
- EPI Expanded Programme on Immunization
- GCP Good Clinical Practices
- GMT Geometric Mean Tire
- IB Investigator Brochure
- ICF Informed Consent Form
- ICH International Conference on Harmonization
- IEAG Indian Expert Advisory Group for Polio Eradication
- IEC Independent Ethics Committee
- LAR Legally Acceptable Representative
- mOPV1 Monovalent Oral Poliomyelitis Vaccine Type 1
- mOPV2 Monovalent Oral Poliomyelitis Vaccine Type 2
- mOPV3 Monovalent Oral Poliomyelitis Vaccine Type 3
- SIAs Supplementary Immunization Activities
- sIgA Secretory Immunoglobulin A
- SOPs Standard Operating Procedures
- TCID Tissue Culture Infective Dose
- tOPV Trivalent Oral Poliomyelitis Vaccine
- VAPP Vaccine-associated paralytic poliomyelitis.
- WHO World Health Organization

**TABLE OF CONTENTS**

[Preamble](#__RefHeading___Toc189285216) 6

[Background](#__RefHeading___Toc189285217) 7

[Name, and description of products:](#__RefHeading___Toc189285218) 7

[Summary of findings from non-clinical studies:](#__RefHeading___Toc189285219) 7

**TRIAL DESIGN**

JUSTIFICATION BIVALENT OPV (bOPV)……..………………………………………………………8

SUMMARY OF KNOWN AND POTENTIAL RISKS….………………………………………..……....9

[Description and justification for the route of administration, dosage, dosage regimen, and treatment period:](#__RefHeading___Toc189285220) 9

[A statement that the trial will be conducted in compliance with protocol, Good Clinical Practice (GCP), and the applicable regulatory requirements:](#__RefHeading___Toc189285221) 10

[Description of population to be studied:](#__RefHeading___Toc189285222) 10

[Trial Objectives and Purpose](#__RefHeading___Toc189285224) 10

[Humoral immunity:](#__RefHeading___Toc189285225) 11

[Safety:](#__RefHeading___Toc189285226) 12

[Trial Design](#__RefHeading___Toc189285227) 12

[A specific statement of primary and secondary endpoints:](#__RefHeading___Toc189285228) 13

[A description of what kind of trial:](#__RefHeading___Toc189285229) 14

[A description of the measures taken to minimize/avoid bias, including randomization & blinding:](#__RefHeading___Toc189285230) 14

[A description of trial treatment, dosage, dosage regiment of the investigational product, dosage form, packaging and labelling:](#__RefHeading___Toc189285231) 14

[The expected duration of subject participation, and a description of the sequence and duration of all trial periods, including follow-up:](#__RefHeading___Toc189285232) 14

[A description of "stopping rules" or "discontinuation criteria" for individual subjects, parts of the trial and the entire trial:](#__RefHeading___Toc189285233) 15

[Accountability procedures for the investigational product, including the comparator:](#__RefHeading___Toc189285234) 15

[Maintenance of treatment randomization codes and procedures for breaking codes:](#__RefHeading___Toc189285235) 16

[The identification of any data to be recorded directly on the CRFs (i.e. no prior written or electronic record of data), and to be considered to be the source data:](#__RefHeading___Toc189285236) 16

[Selection and Withdrawal of Subjects](#__RefHeading___Toc189285237) 16

[Subject inclusion criteria:](#__RefHeading___Toc189285238) 16

[Subject exclusion criteria:](#__RefHeading___Toc189285239) 16

[Subject withdrawal criteria (terminating investigational product treatment/trial treatment) and procedures specifying 1) when and how to withdraw subjects; 2) the type and timing of the data to be collected for withdrawn subjects; 3) whether and how subjects are to be replaced; 4) the follow-up for subjects withdrawn from investigational product treatment/trial treatment:](#__RefHeading___Toc189285240) 17

[Treatment of Subjects](#__RefHeading___Toc189285241) 17

[The treatment to be administered, including the name of all products, the dose, the dosing schedule, the route/mode of administration, and the treatment periods, including follow-up](#__RefHeading___Toc189285242)…….17

[Medications/treatments permitted (including rescue medication) and not permitted before and/or during the trial:](#__RefHeading___Toc189285243) 18

[Procedures to monitor subject compliance:](#__RefHeading___Toc189285244) 18

[Assessment of Efficacy](#__RefHeading___Toc189285245) 17

[Specification of the efficacy parameters:](#__RefHeading___Toc189285246) 18

[Methods and timing for assessing, recording, and analyzing the efficacy parameters:](#__RefHeading___Toc189285247) 18

[Assessment of Safety](#__RefHeading___Toc189285248) 20

[Specification of the safety parameters:](#__RefHeading___Toc189285249) 20

[The methods and timing for assessing, recording, and analyzing safety parameters:](#__RefHeading___Toc189285250) 20

[Procedures for eliciting reports of and for recording adverse event and intercurrent illnesses:](#__RefHeading___Toc189285251) 20

[The type and duration of follow-up of subjects after adverse events:](#__RefHeading___Toc189285252) 20

[Statistics](#__RefHeading___Toc189285253) 20

[A description of the statistical methods to be employed, including timing of any planned interim analysis:](#__RefHeading___Toc189285254) 20

[the number of subjects to be enrolled, reason for choice of sample size, including reflections on (or calculation of) the power of the trial and clinical justification:](#__RefHeading___Toc189285255) 21

[the level of significance to be used:](#__RefHeading___Toc189285257) 21

[criteria for termination of the trial:](#__RefHeading___Toc189285258) 22

[procedures for accounting for missing, unused, and spurious data:](#__RefHeading___Toc189285259) 22

[procedures for reporting any deviation from the original statistical plan:](#__RefHeading___Toc189285260) 22

[the selection of subjects to be included in the analysis (e.g., all randomized subjects, all doses subjects, all eligible subjects, evaluable subjects):](#__RefHeading___Toc189285261) 22

[Direct Access to Source Data/Documents](#__RefHeading___Toc189285262) 22

[Quality Control and Quality Assurance Procedures](#__RefHeading___Toc189285263) 23

[Study Monitoring and Source Data Verification](#__RefHeading___Toc189285264) [Auditing](#__RefHeading___Toc189285265) 23

[Ethics](#__RefHeading___Toc189285266) 25

[Data Handling and Record Keeping](#__RefHeading___Toc189285267) 25

ROLE OF WHO……………………………………………………………………………… …25

[Insurance](#__RefHeading___Toc189285268) 27

[Publication policy](#__RefHeading___Toc189285269) 27

[References:](#__RefHeading___Toc189285270) 28

SUPPLEMENTS………………………………………………………………………………...................................**30**

**ANNEX I**……………………………………………………………………………………………………………...**31**

[ANNEX II](#__RefHeading___Toc189285271) 32

[ANNEX III](#__RefHeading___Toc189285273) 33 - 37

[ANNEX IV](#__RefHeading___Toc189285274) 38 -41

41[ANNEX V](#__RefHeading___Toc189285274) 42 - 49

[ANNEX VI](#__RefHeading___Toc189285274) 50 - 55

[ANNEX VII](#__RefHeading___Toc189285274) 56 - 59

[ANNEX VIII](#__RefHeading___Toc189285274) 60 - 63

[**ANNEX IX…………………………………………………………………………………………………… .64**](#__RefHeading___Toc189285274) **- 68**

**Preamble**

With polio eradication making rapid progress, India is one of the four remaining polio-endemic countries and poses a unique challenge for global eradication due to extremely efficient poliovirus transmission . Until April 2005, the Polio Eradication Initiative in India, and globally, has relied exclusively on trivalent oral poliovirus vaccine (tOPV). In 2005, first monovalent type one oral poliovirus vaccine (mOPV1) and then mOPV3 were used for the first time in large-scale campaigns in India to boost humoral & intestinal immunity. In 2009, a new bivalent (type 1+3) OPV (bOPV) was licensed in India, and will be used extensively in the country in 2010, following the recommendations of the Advisory Committee on Polio Eradication (ACPE) [1].

A trial conducted during May 2009 in Moradabad district, Uttar Pradesh, India, showed that > 99% of children 6-9 months of age had detectable antibodies against poliovirus type 1. These results suggest that the strategy of providing mOPV1 through monthly immunization rounds with high coverage is successfully achieving high population immunity. However, these results raise the question of why this high level of population immunity did not interrupt the remaining chains of poliovirus transmission, and children still get infected with wild poliovirus in Uttar Pradesh. Is it possible that insufficient or waning mucosal immunity allows transmission of poliovirus among children who are otherwise protected against paralysis? And, can we boost mucosal immunity in these children to decrease the circulation of virus?

The proposed trial will evaluate mucosal immunity before and after receipt of two different poliovirus vaccine types, in children living in areas at high risk for poliovirus infection. This trial will be conducted after approval from the Indian Regulatory Authorities, Drugs Controller General of India [DCG(I)], and the relevant ethical review committees.

# Background

## Name and description of products:

1. Bivalent type 1 + 3 oral poliovirus vaccine (bOPV) containing at least 10^6^ TCID_50_ of Sabin poliovirus type 1 and at least 10^5.8^ TCID_50_ of Sabin poliovirus type 3, respectively, produced from imported bulks by Panacea Biotec Ltd, New Delhi, India.

2. Inactivated poliovirus vaccine (IPV) formulated to contain 40-8-32 D-antigen potency, produced from imported blended bulks by Panacea Biotec Ltd, New Delhi, India.

## Summary of findings from non-clinical studies:

IPV has been used extensively since 12 April 1955, when it was first licensed in the United States after a large field trial demonstrated its efficacy [2, 3]. In the 1960s, an enhanced-potency IPV was formulated that has become the standard formulation used today. Approximately 80 million doses of IPV are used each year, primarily in industrialized countries. The safety profile of IPV is well-characterized, and no serious adverse events have been associated with IPV use. Similarly, the immunogenicity of IPV is excellent with seroconversion rates approaching 100% if administered outside the window with the highest levels of maternally-derived antibody (birth-2 months of age) [2]. In addition, a single dose of IPV following multiple doses of tOPV has been shown to close the remaining immunity gaps very reliably in developing countries [4-6].

bOPV vaccine has been licensed recently in 2009 in Belgium and India. It has been assumed that the efficacy of bOPV would be similar or higher than that of tOPV, but only ~ 300,000 doses were used in Estonia, Lithuania, and Hungary during 1959-1960 [7]. A clinical trial conducted in India during 2009 demonstrated that for both serotypes (1 and 3), bOPV was superior to tOPV and non-inferior to the respective monovalent OPVs [8]. At present the risk of vaccine-associated paralytic poliomyelitis (VAPP) following administration of bOPV is unknown, but assumed to be lower than the risk following tOPV because of the absence of the Sabin Type 2 strain, which is the major cause of VAPP among immunodeficient individuals.

These two vaccines are produced by Panacea Biotec Ltd. and are licensed for use in India by the Drug Controller General of India [DCG(I)].

Trial design justification:

This trial calls for a three-arm design (IPV, bOPV, or unvaccinated) in three age groups, infants aged 6-11 months, children aged 5-6 years, and children aged 10-11 years. A challenge dose with bOPV will be administered to all study subjects to assess mucosal immunity.

This design will provide the first data on mucosal immunity in children living in high risk areas in India. This trial will also assess which polio vaccines are more efficient in boosting mucosal immunity, which would provide an effective barrier to subsequent excretion and community spread of poliovirus. The data from this trial will likely have programmatic implications, especially in determining whether the current age range for SIAs (children aged <5 years) should be re-evaluated. Additionally, the trial will compare different markers of mucosal immunity such as measurement of secretory immunoglobulin A (sIgA) levels in gingival fluid or stools and ELISPOT test, with the gold standard test, which is the measurement of vaccine virus shedding after a challenge with a known dose of OPV. Identifying new surrogate markers of mucosal immunity is important because the challenge test requires high workload and resources, and its use in the post-eradication era may not be possible.

Summary of known and potential risks:

The main serious adverse event associated with oral poliovirus vaccines, including bOPV, is VAPP. Outside the first few months of life, the risk of VAPP is small; approximately 1 case per 1 million doses of tOPV administrated [9, 10], and associated primarily with type 3 and type 2 components of the tOPV. Since bOPV does not contain the Sabin type 2 component, we can assume that the already very low risk of VAPP is even lower following bOPV administration. However, the exact risk of VAPP following bOPV is currently unknown. The only experience with bOPV is available from Estonia, Lithuania and Hungary from 1959-1960. Here the experience is mixed, while Estonia and Lithuania reported no VAPP [10-14], Hungary reported an increase in VAPP [15-17]. The other potentially serious adverse events are anaphylactic reactions. Since OPV and IPV vaccines contain trace amounts of antibiotics, anaphylactic reactions to these antibiotics are possible, but appear to be exceedingly rare. IPV is one of the safest available vaccines, and no systemic serious adverse events are linked to this product. As with all injectable vaccines, minor local adverse events at the injection site are relative frequent.

## Description and justification for the route of administration, dosage, dosage regimen, and treatment period:

At study entry (day 0), subjects will be randomized to one of three study arms. Arm A will be vaccinated with one dose of bOPV administered by the oral route at day 0; arm B will receive one dose of IPV intramuscularly; and arm C will not receive any vaccination. At day 28, all subjects will receive a single dose of bOPV that serves as a challenge dose.

A study from India [18] and a review of mOPV clinical studies is available [19].

## A statement that the trial will be conducted in compliance with protocol, Good Clinical Practice (GCP), and the applicable regulatory requirements:

The study will be conducted in compliance with this protocol, Good Clinical Practice, and all applicable regulatory requirements.

Description of population to be studied:

Healthy children aged 6-11 months, 5-6 years and 10-11 years, who live within a relatively short and easily accessible distance (<30 km) from a study clinic, and do not plan to travel away during the entire 2-month study period.

## References to literature and data relevant to trial, and that provide background for the trial:

The Advisory Committee on Poliomyelitis Eradication (ACPE)

The ACPE has endorsed the need for this trial during the 18-19 November 2009 meeting in Geneva [1].

Polio Research Committee (PRC)

The PRC has reviewed the study protocol in detail during the 10-11 November 2009 meeting in Geneva, has endorsed the study protocol and approved funding.

# Trial Objectives and Purpose

## Mucosal immunity:

- To compare baseline mucosal immunity (through different methods) among three age groups with several degrees of protection acquired through immunization:
  - Infants 6-11 months of age will have received a few OPV doses
  - Children 5-6 years of age will have received multiple OPV doses in campaigns and are expected to have the highest levels of mucosal and humoral immunity
  - Children 10-11 years of age will not have received booster OPV doses for > 5 years. They are expected to have high antibody titers but may have waned mucosal immunity.
- To determine whether one dose of bOPV or IPV significantly reduces excretion of poliovirus types 1 and 3, respectively, following a challenge dose with bOPV, compared to an unvaccinated control arm.
- To determine whether one dose of bOPV or IPV significantly increases sIgA in crevicular fluids and stool samples, and to examine whether the increase in specific SIgA in these specimens is correlated to a reduction in excretion of Sabin poliovirus types 1 and 3 following the challenge with bOPV. This will allow evaluating the use of sIgA as a surrogate test for assessment of mucosal immunity.
- To determine the robustness of the mucosal immune response by study arm and age group, by measuring poliovirus antibody-secreting cells in an ELISPOT-spot assay 7 days after the administration of the study vaccine.

Humoral immunity:

- To determine whether one dose of bOPV or IPV will significantly increase seroprevalence and booster antibody titers compared to unvaccinated children.
- To assess whether one dose of IPV will significantly increase seroprevalence and antibody titers compared to a single dose of bOPV.
- To examine the correlations between antibody titers in blood and shedding of poliovirus after a challenge dose, for each vaccine study arm and age group.
- To examine the correlation between levels of IgA in serum and levels of secretory IgA in crevicular fluids and stool samples, before and after the booster vaccine dose, to gain further understanding on the mechanisms of mucosal immunity against poliovirus.
- To analyze correlations between levels of secretory IgA in crevicular fluids and stools and the ELISPOT test results, to better understand mucosal immunity against poliovirus.

Safety:

- To assess adverse events following vaccine administration.

# Trial Design

Local community mobilization coordinators (CMCs) working with the Global Polio Eradication Initiative (GPEI) will identify families with children in the target age-group (i.e. 6-11 months, 5-6-years, and 10-11-years). Surveillance Medical Officers (SMOs) will visit households of potential candidate families, explain the study to the parents or guardians, and facilitate transportation to one of the study sites for those interested to participate. Approximately 10 study sites will be chosen in areas identified as high-risk for poliovirus transmission.

At the study site, a physician will conduct a short interview and physical examination. After obtaining informed consent from the parents (and assent from children of age 10-11 years), the study participant will be assigned a study number and allocated to a study arm. For this, the parent or child will select a closed envelope that includes the study arm from a basket. Then a baseline blood sample collected by venipuncture, oral (crevicular) fluid, and stool samples will be collected before vaccine administration.

Note: crevicular fluid will not be collected in the 6-11 month age group because of the absence of teeth.

In a subset of children 10-11 years of age (~175-200 children) a second blood sample will be collected at 7 days by venipuncture for the ELISPOT test.

In all children, at 28 days, another blood sample by venipuncture, another oral (crevicular) fluid sample and a stool sample will be collected before the subjects receives the challenge vaccine (bOPV). Thereafter, at days 3, 7 and 14 stool samples will be collected. And finally, at day 56, another serum sample and oral fluid sample will be collected. At this time, having fulfilled the study requirements, the children will exit the study.

## A diagram of the study design is shown below.

## A specific statement of primary and secondary endpoints:

The primary endpoint is reduction in excretion at days 3,7 and 14 post- challenge dose of vaccine poliovirus administered 28 days after a single dose of bOPV or IPV.

The secondary endpoints are:

- Increase of sIgA in gingival fluids and stools 28 days after a dose of bOPV or IPV.
- Increase in poliovirus antibody secreting cells (measured with ELISPOT) 7 days following a dose of bOPV or IPV.
- Seroconversion or antibody titer boosting 28 days following a single dose of bOPV or IPV.

A description of what kind of trial:

This is a randomized controlled unblinded trial.

## A description of the measures taken to minimize/avoid bias, including randomization & blinding:

Since the vaccines are administered by different routes (oral for bOPV, and intramuscular for IPV), it is not possible to blind the investigators and the study staff (doctors, nurses, statisticians). However, all serum, oral fluid, and stool samples will be blinded to the laboratory investigators.

## A description of trial treatment, dosage, dosage regimen of the investigational product, dosage form, packaging and labelling:

## A description of trial treatment, dosage, dosage regimen of the investigational product and dosage form is provided above.

## IPV (bulk source: NVI, Netherlands) and bOPV (bulk source: PT Biofarma) of Panacea Biotec Ltd. will be made available in the commercial packaging. IPV will be made available as single dose prefilled syringe and bOPV will be supplied as multidose vial for oral use. The batch numbers, expiration dates and physical description will be present on the label.

## Additional information will be added as given-

| FOR CLINICAL TRIAL PURPOSE ONLY |
| --- |
| Protocol Number: PBL/CR/2011/02/CT |
| Sponsor - Panacea Biotec Ltd. |

## The sponsor will supply the study vaccines. The investigator (or pharmacist) will make an inventory and acknowledge receipt of all shipments of study vaccines. All vaccines are to be stored in accordance with the instructions of the manufacturer.

The expected duration of subject participation, and a description of the sequence and duration of all trial periods, including follow-up:

The study participants will be enrolled for a total of ~60 days. No additional follow-up is planned.

## A description of "stopping rules" or "discontinuation criteria" for individual subjects, parts of the trial and the entire trial:

No stopping rules or discontinuation criteria appear necessary or are envisioned. The trial will be monitored by the Data and Safety Monitoring Board (DSMB), and serious adverse events will be reported according to GCP requirements to the sponsor within 24 hours, and to the respective regulatory agencies and ethical review committees within 15 days.

## Accountability procedures for the investigational product, including the comparator:

Comprehensive training of all study staff, and a detailed questionnaire will ensure and document that study protocol requirements are being followed. Vaccine will be stored according to cold chain requirements, and detailed inventory logs will be maintained.

## Maintenance of treatment randomization codes and procedures for breaking codes:

This trial is not blinded.

## The identification of any data to be recorded directly on the CRFs (i.e. no prior written or electronic record of data), and to be considered to be the source data:

A questionnaire (Annex IV) will serve as the primary data collection instrument, and will serve as the source data.

# Selection and Withdrawal of Subjects

All eligible children within the correct age group in the study area will be eligible to participate. Study participants may withdraw for any reason at any time. Withdrawal will not affect in any way the treatment of the child in the study institution.

## Subject inclusion criteria:

Healthy children aged 6-11 months, 5-6 years or 10-11-years, who reside within a relatively short and easily accessible distance (<30 km) to the study sites, and do not plan to travel away during entire the study period (~2 months).

## Subject exclusion criteria:

Children with known thrombocytopenia or bleeding disorders; children acutely ill or with signs of acute infection (e.g. fever ≥ 101 F) at the time of enrolment; residence >30 km from study site; or families expecting to be absent during the 60-day study period. A diagnosis or suspicion of immunodeficiency disorder (either in the participant or in a member of the immediate family) will also render the child ineligible for the study.

## Subject withdrawal criteria (terminating investigational product treatment/trial treatment) and procedures specifying 1) when and how to withdraw subjects; 2) the type and timing of the data to be collected for withdrawn subjects; 3) whether and how subjects are to be replaced; 4) the follow-up for subjects withdrawn from investigational product treatment/trial treatment:

As mentioned above, subjects may withdraw from the study at any point. The data collected for withdrawn subjects, in addition to standard questionnaire data, will include the reason for withdrawal. Subjects will not be replaced. No additional follow-up is envisioned for withdrawn subjects. If children miss a scheduled blood sample collection, they will be excluded from final analysis. Children missing a stool or gingival fluid sample but having other samples available will be included in the study. If children develop an acute illness during the study, medical treatment will be provided as explained on page 20, and sample collection will continue as scheduled, unless the illness is associated with a contraindication for venepuncture or the parents refuse sample collection. In this case, the study subject will be withdrawn from the study as explained above.

# Treatment of Subjects

## The treatment to be administered, including the name of all products, the dose, the dosing schedule, the route/mode of administration, and the treatment periods, including follow-up:

See above.

## Medications/treatments permitted (including rescue medication) and not permitted before and/or during the trial:

There will be no restrictions in using medications/treatments. A diagnosis of a B-cell immunodeficiency disorder would lead to exclusion from the study.

## Procedures to monitor subject compliance:

All interventions will be conducted by study personnel; the only subject compliance required is to abide by scheduled visits.

# Assessment of Efficacy

## Specification of the efficacy parameters:

Humoral immunity: Sera collected at study entry (day 0) and during the 7-, 28- and 56-day visits, will be examined for the presence (detectable) or absence of neutralizing antibodies to all three poliovirus serotypes. A reciprocal titer of ≥8 is considered to indicate the presence of neutralizing antibodies. In addition, a change from non-detectable (reciprocal <8) to detectable (reciprocal >8), or from below the highest dilution tested (reciprocal <1448) to the highest dilution tested (reciprocal >1448) will also indicate seroconversion. Although highest titre for neutralizing antibodies is 1024, we are taking end point dilution of half a log higher then 1024. This log of 10.5 is the highest observable titre and is being used because of high titres observed in this highly immunized population.

For participants with detectable antibodies, antibody boosting is defined as a >4-fold increase.

Mucosal immunity: Stool specimens will be collected at 0, 28, 31, 35 and 42 day visits. The samples will be examined for the presence or absence of polioviruses and other enteroviruses. In positive samples, the virus type will be determined following standard protocols from the World Health Organization (WHO) [20]. In addition, the amount (titration) of virus particles will be measured in those samples positive for poliovirus.

Semi-quantitative levels of sIgA in gingival fluid and stool samples will be determined using ELISA. High, medium and low levels will be determined and correlated in each study subject with serum antibody levels and with virus shedding after the challenge (Annex VIII-IX).

Additionally, in a subset of children 10-11 years of age, blood samples collected on days 0 and 7 will be tested to detect the amount of antibody-secreting lymphocytes specific for poliovirus using the ELISPOT technique.

## Methods and timing for assessing, recording, and analyzing the efficacy parameters:

Humoral immunity: The sera will be processed for the determination of neutralizing IgG titers to poliovirus types 1, 2 and 3, using a modified neutralization assay according to a standard protocol (Annex VI). To ensure reproducibility, the neutralization assays for each participant will be run on the same plate. Both positive and negative controls will be used. All samples will be tested in triplicate. This test will be performed in the Enterovirus Research Center in Mumbai, supervised by the principal investigator.

Serum IgA for poliovirus types 1, 2 and 3 will also be tested in the Enterovirus laboratory at the Centers for Disease Control and Prevention (CDC) using ELISA (protocol in Annex VII).

Mucosal immunity: The stool samples will be processed in the laboratories in India from the Global Poliovirus Laboratory Network, according to the standard protocol used in all network laboratories, which includes isolation, intratypic differentiation (ITD), and sequencing of polioviruses. All network laboratories are accredited each year by WHO, a process that involve an on-site inspection, proficiency testing, etc. [20].

The samples from crevicular fluid and an aliquot of each stool sample will be sent to the Enterovirus laboratory of the Centers for Disease Control and Prevention (CDC). Determination of sIgA in crevicular fluids and stool will be performed in the CDC laboratory because of the current absence of standardized protocols and technical accreditation in Indian laboratories. The protocols for both tests are included in Annex VII and VIII.

The ELISPOT testing will be done according to a standard protocol developed by the International Vaccine Institute (IVI) Korea [21] at the National Institute of Cholera and Enteric Diseases (NICED), Kolkata, India. NICED is an institute under Indian Council of Medical Research (ICMR), Government of India. The protocol for this testing is included in Annex X.

# Assessment of Safety

## Specification of the safety parameters:

All participants will be informed to contact the study personnel if the child requires medical care. Should a serious illness occur while enrolled in the study (requiring a physician's visit or hospitalization), parents will receive instructions on whom to contact within the study institutions.

## The methods and timing for assessing, recording, and analyzing safety parameters:

The study questionnaire will be used to record data during each visit, and for the preceding period, if applicable.

## Procedures for eliciting reports of and for recording adverse event and intercurrent illnesses:

As discussed above, parents will be encouraged to use the study institution for medical services.

## The type and duration of follow-up of subjects after adverse events:

Should adverse events occur, referral and medical care will be provided.

# Statistics

## A description of the statistical methods to be employed, including timing of any planned interim analysis:

A statistical analysis plan is provided in Annex II

**Humoral immunity**: Chi-square to compare the proportion of children seropositive in each study arm and age group; non-parametric tests to compare the titer distribution among the study arms.

**Mucosal immunity:** Chi-square to compare the proportion of subjects excreting poliovirus type 1, type 2, and type 3 at each collection point among study arms and age-groups; non-parametric tests to compare the median titers of poliovirus shed by individuals at each study arm and age-group.

## The number of subjects to be enrolled, reason for choice of sample size, including reflections on (or calculation of) the power of the trial and clinical justification:

## We estimate a required sample size of at least 801 children to complete the 2-month study (89 per study arm and age group). To account for an estimated drop-out of 15-20%, a total of 990 evaluable children will be enrolled in each arm (110 per age group and study arm). Calculations and justification for sample size estimates are provided in Annex I.

## The level of significance to be used:

We are seeking to show (a) a reduction in number of subjects excreting vaccine poliovirus following a challenge vaccine dose (bOPV) among those vaccinated with bOPV or IPV compared to the control group; (b) an increase in secretory IgA following vaccination with bOPV or IPV compared to a control group; and (c) an increase in seroprevalence or antibody titers following vaccination with bOPV or IPV compared to a control group.

We use a level of significance of 0.05 (two-tailed test) and a power of 90% to detect the │20%│difference in proportion of subjects shedding vaccine poliovirus after the challenge dose of bOPV(see annex I).

## Criteria for termination of the trial:

The trial will be terminated if so directed by the Data and Safety Monitoring Board (DSMB) or other applicable agencies following safety concerns, or once sufficient numbers of study subjects have completed the study requirements.

## Procedures for accounting for missing, unused, and spurious data:

We will do "Modified Intention to Treat Analysis". All evaluable subjects will be included in the analysis. Evaluable subjects will mean the subjects who complete all study requirements including all study visits and provide sufficient quantities of sera samples for analysis. The subjects where sera are available, but missing a stool or gingival fluid sample, will also be included in the analysis.

## Procedures for reporting any deviation from the original statistical plan:

Additional analysis may be required, and will be conducted if agreed among the participating institutions.

## The selection of subjects to be included in the analysis (e.g., all randomized subjects, all doses subjects, all eligible subjects, evaluable subjects):

As stated above, only subjects who complete all study requirements including all study visits and provide sufficient quantities of sera samples for analysis will be included in the analysis.

# Direct Access to Source Data/Documents

The investigator/institutions will permit (by way of written agreement) trial-related monitoring, audits, IRB/IEC review, and regulatory inspection, providing direct access to source data/documents.

# Quality Control and Quality Assurance Procedures

### Study Monitoring and Source Data Verification

After appropriate ethical approval by an Institutional Review Board (IRB) is available (and the final protocol has been amended as required by IRB), the field study clinics will be set up to ensure the presence of all logistics. All study staff will be given detailed training on the study and their specific role. During the set-up of study sites and trainings, the requirements of GCP, protocol procedures, and all logistical issues will be discussed at length. The subjects cannot be enrolled until occurrence of these processes and their documentation.

After the study is initiated, the study monitor will be in regular contact with the sites to obtain information on the performance of the study. These contacts will be scheduled to take place at regular intervals. Subsequent to start of recruitment, routine monitoring visits would occur (approximately every 4 weeks) after prior appointment with the investigators.

The investigator and his/her staff are obliged to devote a suitable amount of time and an appropriate place for the monitoring visits. During each visit, the monitor will review the Case Report Form (CRF) of each subject in the study with regard to completeness, thoroughness and compliance with the protocol. In addition, at a minimum, the original subject data (e.g., entry cards, index cards, original findings) will be reviewed to ensure that:

- subject informed consent is incorporated;
- inclusion/exclusion criteria are properly followed;
- the CRF data are consistent with the questionnaires, the source data;
- all relevant clinical and laboratory findings and concomitant medication are documented in the CRFs;
- quantity and dosing schedule of concomitant medication is documented in the CRFs;
- quantity and dosing schedule of the Investigational/Comparator Product is in accordance with the protocol;
- all relevant information (e.g., any adverse event) has been recorded in the appropriate place in the CRFs;
- the Investigational/Comparator Product is being stored correctly, and its supply is being properly accounted for;
- Incorrect or illegible entries in the CRFs would be submitted to the investigator for correction.

The monitor will retrieve completed CRFs during the regularly held monitoring visits.

During the study trial period, the responsible Panacea Biotec staff will be available to answer questions with regard to the performance of the study.

### Auditing

In addition to the above outlined monitoring visits, the participating institutions may be audited. This audit may be carried out by representatives of Panacea Biotec or by the responsible regulatory authority(ies). Such an audit would be done to review whether the data has been properly recorded in the interim or final report and whether the performance of the study is in accordance with the protocol, the standard operating procedures (SOPs) developed for the study, and other relevant guidelines. Subject confidentiality will be maintained at all times.

Panacea Biotec Ltd. will immediately inform the investigators if an audit has been requested by a regulatory authority.

# Ethics

The study protocol will be reviewed and approved by the Ethical review committee (ERC) at WHO/HQ and by the Institutional Review Board of the Indian Council Medical Research. The study will be approved for implementation by the Drug Controller General (India).

# Data Handling and Record Keeping

The questionnaire data will be double-entered into an electronic data file; the laboratory data will be provided in electronic form and merged based on a common identifier. The original questionnaires will be stored with the Sponsor. Copies of all study documentation, including the questionnaires will be sent to WHO for long-term storage.

**Description of the roles and responsibilities of the institutions participating**

1. **WHO** will be a funding partner, and has an interest to ensure that the highest possible standards are applied, and that the trial will produce data that will be accepted not only in India, but by regulatory authorities elsewhere. In addition, contribution of WHO will include:
   - 1. Design the study and monitor implementation through visits to the study sites
     2. WHO will provide partial funding (as discussed during the licensing process). Funding would be made available through a technical services agreement to the study site(s) and the laboratory. Funding will include contracting healthcare workers involved in study procedures (physicians, vaccinators, laboratory technicians and others).
     3. SMOs from the National Polio Eradication Program (WHO) will enroll study patients, conduct data collection, supervise adherence to study protocols and standard operating procedures, and monitor processing, storage and transportation of specimens.
     4. Several laboratories from the WHO polio laboratory network in India will process blood and stool samples.
     5. WHO will receive the final clean data set (questionnaire + laboratory data) as soon as available and review report(s) and articles for publication.
2. **The Indian Council for Medical Research** will act as coordinator of the study in India, ensuring quality and safety of study procedures and data collection. The principal investigator, Dr. Jagadish Deshpande works for the ICMR.
3. **CDC** will provide technical assistance in development of protocol and standard operating procedures, training, statistical analysis, and interpretation of results. CDC will also supervise training, implementation of study procedures and data collection. A CDC laboratory will process isolates from blood, crevicular and stool samples for additional tests measuring antibodies against poliovirus and for quality control of tests conducted in the Indian laboratories.
4. **Panacea Biotec Ltd** assumes roles and responsibilities of the sponsor of the study, including study insurance.
5. **The Ministry of Health and Family Welfare of India** will provide approvals for the study to be conducted and ensure the support of state health authorities.
6. The **Department of Health and Family Welfare of Uttar Pradesh** will facilitate the implementation of the study by allowing the use of public health care facilities as study sites. Health care workers in Public Hospitals or Public Health Care facilities may also need to see and counsel study patients who seek care after vaccine administration when study staff is not available.
7. **The International Vaccine Institute (IVI) Korea** will conduct the ELISPOT testing procedures.
8. **Imperial College, London will assist with high level statistical analysis.**

# Insurance

Panacea Biotec Ltd has umbrella insurance for clinical trials. This insurance will provide coverage for medical care in the event an adverse event occurs.

# Publication policy

All publications emanating from this trial will be reviewed by the participating institutions. Authors will be determined based on actual input into the publications, according to existing guidelines.

**REFERENCES:**

1. WHO. Conclusions and recommendations of the Advisory Committee on Poliomyelitis Eradication, November 2009. Special consultation with polio-infected countries and global management team partners, Geneva, 18-19 November 2009. Wkly Epidemiol Rec 2010;85:1-11

2. Plotkin SA, Vidor E. Poliovirus Vaccine - Inactivated. In: Plotkin SA, Orenstein WA and Offit PA, eds. Vaccines. 5th ed. Philadelphia: Saunders, 2008:605-630

3. Robbins FC. The history of polio vaccine development. In: Plotkin S, Orenstein WA, eds. Vaccines. 4th ed. Philadelphia, PA: W.B. Saunders, 2004

4. Hanlon P, Hanlon L, Marsh V, et al. Serological comparisons of approaches to polio vaccination in the Gambia Lancet 1987;1:800-1

5. Moriniere BJ, van Loon FP, Rhodes PH, et al. Immunogenicity of a supplemental dose of oral versus inactivated poliovirus vaccine Lancet 1993;341:1545-1550

6. Sutter RW, Suleiman AJ, Malankar PG, et al. Sequential use of inactivated poliovirus vaccine followed by oral poliovirus vaccine in Oman. J Infect Dis 1997;175 Suppl 1:S235-40

7. Parkman PD. An assessment of the safety and efficacy implications of removing the type 2 strain trom the trivalent oral poliovirus vaccine. Vaccine Res 1997;6:49-66

8. Sutter RW, John TJ, Jain H, Agarkhedkar S, Ramanan PV, Verma H, Deshpande J, Singh AP, Sreevatsava M, Malankar P, Burton A, Chatterjee A, Jafari H, Aylward RB. Randomized Clinical Trial of bivalent Type 1 and 3 Oral Poliovirus Vaccine. Lancet 6736(10)61230-5

9. Strebel PM, Sutter RW, Cochi SL, et al. Epidemiology of poliomyelitis in the United States one decade after the last reported case of indigenous wild virus-associated disease. Clin Infect Dis 1992;14:568-79

10. Sutter RW, Kew OM and Cochi SL. Poliovirus vaccine - live. In: Plotkin SA, Orenstein WA and Offit PA, eds. Vaccines. Philadelphia: Saunders, 2008:631-685

11. Drosdov SG, Raudam EJ, Tapupere VO, Shirman GA, Kuslap TP and Knyazeva TV. Laboratory investigation of poliomyelitis cases reported in areas of mass immunization of the population in the Estonian S.S.R. with live poliovirus vaccine. In: Chumakov MP, ed. Oral Live Poliovirus Vaccine. Moscow, 1961:52-58

12. Podsedlovsky TS, Uspensky YS and Chumakov MP. Some results of mass immunization with live poliovirus vaccine inthe Lithuanian S.S.R., 1959. In: Chumakov MP, ed. Oral Live Poliovirus Vaccine. Moscow, 1961:59-64

13. Vasilyeva KA. Some results of mass poliomyelitis immunization with live attenuated vaccine from Sabin strains in the Estonia S.S.R. In: Chumakov MP, ed. Oral Live Poliovirus Vaccine. Moscow, 1961:47-51

14. Dobrova IN, Yankevich OD, Voroshilova MK and Chumakov MP. Serologic evaluation of various schedules of oral immunization. ? 1961:313-316

15. Domok I. Experiences associated with the use of live poliovirus vaccine in Hungary, 1959-1982. Rev Infect Dis 1984;6 Suppl 2:S413-8

16. Domok I, Baranyai E, Kovacs F, Karasszon D, Lengyel GY and Szollosy E. Clinical and virological analysis of case diagnosed as poliomyelitis in Hungary in the years 1961-1967. In: Proceedings 12th European Symposium of Poliomyelitis and Allied Diseases. Bucharest, 1969

17. Weiszfeiler J, Fornosi F, Katay A, Domok I, Rudnay O and Boda D. Vaccination against poliomyelitis with live Sabin vaccine in Hungary. In: Chumakov MP, ed. Oral Live Poliovirus Vaccine. Moscow, 1961:38-46

18. John TJ, Devarajan LV and Balasubramanyan A. Immunization in India with trivalent and monovalent oral poliovirus vaccines of enhanced potency. Bull World Health Organ 1976;54:115-7

19. Caceres VM, Sutter RW. Sabin monovalent oral polio vaccines: review of past experiences and their potential use after polio eradication. Clin Infect Dis 2001;33:531-41

20. World Health Organization. Polio laboratory manual. 4th ed. Geneva, 1994.

21. Immunization, Vaccines and Biologicals. Polio laboratory manual. 4th edition. WHO/IVB/04.10. In: WHO ed. Geneva, 2004

22. Janetzki S. Automation of the Elispot technique: past, present and future. JALA 2004;9:10-15

**Supplements**

Annex I - Sample size calculations

Annex II - Analytic plan

Annex III - Informed consent form

Annex IV - Subject information sheet and Assent Form

Annex V - Case Record Form (CRF)

Annex VI - Declaration of Helsinki

Annex VII - Serology protocol

Annex VIII – Antibody-capture ELISA for IgA and IgM to Polioviruses Type 1,2 and 3

Annex IX – Procedure for performing the one-step, dual color Enzyme Linked ImmunoSPOT assay (ELISPOT) for enumeration of Polio-specific IgG and Iga antibody secreting cells (ASC)

# ANNEX I

Sample size calculations

1. We are seeking to demonstrate that excretion of poliovirus following a challenge with bOPV is significantly lower in the bOPV or IPV vaccinated groups compared with the control group.

2. Definitions: (a) Significantly decreased defined as a decrease of ׀20%׀ in the proportion of study subjects vaccinated with IPV or bOPV who excrete virus following a challenge dose of bOPV compared with the proportion of control subjects who excrete virus after the challenge.

3. Applying a power of 0.90 (beta) and an alpha of 0.05, the following samples sizes would be required based upon assumed excretion of control and treatment (IPV or bOPV) groups:

| Control group excretion | bOPV or IPV excretion after challenge dose | | |
| --- | --- | --- | --- |
|  | 0.10 | 0.20 | 0.30 |
| 0.30 | 89 |  |  |
| 0.40 |  | 119 |  |
| 0.50 |  |  | 134 |

5. Using the most likely approach of low excretion in control and treatment groups due to previous exposure to OPV in all subject), we would require a sample of 89 for each study group, for a total of 801 subjects for the 3 study arms and 3 age groups per study arm.

6. With a sample size of at least 89 per study group, we should have a probability of 0.89 or more of estimating any of the differences in excretion of vaccine virus after the challenge dose within a ׀absolute value 20%׀

# 7. To account for drop-outs and ensure that sufficient subjects will complete all study requirements, we will enrol a total of 990 subjects, 110 per age-group (6-11 mo, 5-6 years and 10-11 years) and per study arm (IPV, OPV and controls).

# ANNEX II

Analytic plan

1. The primary endpoint of the study is reduction in excretion of poliovirus following a challenge dose of bOPV in subjects who have received a single dose of bOPV or IPV.

2. We will use chi-square test to compare the proportion of study subjects that excrete virus/or not following the challenge, in each study arm.

3. To use a conservative measure of p, we will apply a two-tailed test. We will also prepare 95% confidence intervals.

4. We will use non-parametric tests to compare the (median) amount of virus excreted after the challenge in each study arm.

5. We will also use non-parametric tests to measure and compare the distribution of antibody titers in serum (only for those with a positive titer; i.e., >1:8), among study groups and before and after treatment.

# ANNEX III

INFORMED CONSENT FORM

***Title of Research Project:***

**ASSESSMENT OF MUCOSAL IMMUNITY TO POLIOVIRUSES AFTER A SUPPLEMENTAL DOSE OF BIVALENT ORAL POLIOVIRUS VACCINE OR INACTIVATED POLIOVIRUS VACCINE IN CHILDREN IN NORTHERN INDIA: A RANDOMIZED TRIAL**

### **Principal Investigator:** Dr. J. Deshpande

Chief Enterovirus Research Center, Indian Council Medical Research.

***Collaborating Institutions:*** Indian Council of Medical Research, Ministry of Health & Family Welfare Government of India, Department of Health & Family Welfare of Uttar Pradesh, and World Health Organization.

**Information Sheet for Parents of Eligible Infants Participating in Research Project**

I am Dr. ___________________ . We are working with the Indian Council of Medical Research, the Ministry of Health & Family Welfare and the Department of Health & Family Welfare of UP. We are doing a study in this area to measure how well two different kinds of poliovirus vaccine work in children in India. Your child has been selected to participate because he (or she) is between 6 months and 11 years of age and he (or she) could benefit from this vaccine.

***Background***

India is very close to polio eradication but we need to give the final push to eradicate polio from UP. In this research study we will compare two new polio vaccines. One is the new oral polio vaccine called bivalent OPV, which is now given in rounds in UP and Bihar. The other vaccine is called inactivated poliovirus vaccine or IPV and it is given in an injection. This injected vaccine is used in routine vaccination against polio in many countries in Europe, Asia and in the Middle East (Saudi Arabia, Jordan, Syria and in the Gulf countries)*.* It is also given in India by private doctors but it is very expensive.

***Purpose of the Study:***

We want to show that an additional dose of the injectable vaccine or the oral polio vaccine is better in reducing the spread of polio in your community.

***Eligibility:***

Your child has been selected because he/she is healthy, and could still be at risk of polio.

***What you will be doing?***

If you decide to participate in the study, your child and a parent will have to come three times to ______________ health facility. The study will arrange transportation for you and your child during all the visits.

During the first visit, we will ask you some questions about yourself and about the child’s health. Then, we will collect a small amount of blood (approximately 1 ml, or 20 drops) from the back of the hand or the arm, and a saliva sample to see if your child already has some protection against polio. The saliva sample will be collected with the help of a swab/device which looks like a tooth brush. It will be required to use this swab for one minute like brushing your teeth and the same will be sent for testing. A fresh new swab will be used for every participant. The procedure is simple, painless and harmless. The saliva sample will not be collected in the 6-11 month age group because of absence of teeth. We will also collect a stool sample and explain the procedure for its collection.

After that, your child will receive 2 drops of oral polio vaccine (bOPV), or an injection with the injectable vaccine (IPV), or nothing. Your child will be placed in each group by chance (like a lottery).

Your child and a parent/guardian will have to go back to the health facility 28 days (4 weeks) later. We will ask you questions about your child’s health, and we will again collect samples of blood, saliva and stools. During this second visit we will also give your child a dose of oral polio vaccine (bOPV). All children will get this dose of vaccine, no matter what they received on the first visit.

After this second visit, you will have to collect stool samples from your child at home. You will have to collect a total of 3 stool samples: 3 days, 7 days, 10 days and 14 days after the second visit. Study staff will give you containers and will explain how to collect and store the stool samples at home. Study staff will also come to your house to pick up the samples.

Then, you will have to go back to the health faclity for the third and final visit, 56 days (8 weeks) after study start. We will again ask questions about your child’s health, and collect blood and saliva. After this visit your participation in the study will be complete.

If your child is 10-11 years of age, we may ask him/her to give a little extra blood on the first visit (3 ml extra, for a total of about half a teaspoon) and to come back 7 days after the first visit for another blood sample of 3 ml. Your child will make a total of four visits for the study, although we will only get extra blood on the first and the second visit.

All these tests will tell us if your child is protected against polio. Also, if you need medical assistance for your child during this time, you can come to ____________ health facility and a physician will examine your child.

**Benefits for the child**

By participating, you will know whether your child is protected against polio and your child will receive extra dose of polio vaccine that will add protection. Your child will also get a free exam by a physician in each visit. Also this study will help your community to see what polio vaccines work better to protect children against polio in India.

To compensate for the time that you or your spouse spent in the health facility for the three/four studies visits, the study will pay the equivalent to one day of wages (200 rupees for the first visit, 250 for the second and300 for the third visit). For those children who have to come one extra time 7 days after the vaccine, they will be paid 350 rupees for the fourth visit. In addition, the study will offer your child a small gift during each visit.

*Risks and Discomforts:*

There is a possible risk of harm to your child with this study, but this risk is not a major one.

Blood drawing may cause discomfort or pain, and local bruising. The blood will be drawn by trained technicians using sterile needles and clean materials.

There will be some pain with the injection. Some children may have redness, swelling, and bruising at the site. The child will receive pain medications if needed. Fever and malaise do not occur with IPV. The vaccine will be given by trained nurses or ANMs (auxiliary nurse midwives) using sterile needles and clean materials.

Since OPV contains very small amounts of antibiotics, an allergic reaction to these antibiotics is possible, but very unlikely.

There is the chance that the child could get a harmful side effect that was not known before the study. If your child suffers any injury from participating in the study, he/she will receive appropriate care and treatment in the district Hospital. The sponsor of this study will bear the cost of this care.

*Confidentiality:*

The names and identity of the parents and their children will be kept private by the investigators and staff of the health facility. Only study personnel that need to keep contact with you and make sure that the samples are properly labeled, will have access to identifying information. Other information without names and identities will be shared with study investigators at the WHO and a committee for watching over the safety of the study.

The blood, saliva and stool samples collected from your child will be used only for the study related tests and will be safely disposed of on completion of the study as per the applicable national guidelines.

No data from this study will be presented, in published format or otherwise, in a manner which could identify your child or any individual participant.

**Results**

Results from your child’s participation in this study will be communicated back to you once they become available.

*Right to refuse or withdraw:*

Your consent for your child to participate in this study is voluntary. You do not have to take part in this study if you do not wish to do so. You may decline participation now or later. If you refuse to participate, your child will not lose any access to medical services or other benefits provided by the government of India and the Government of UP.

The doctors can withdraw your child from the study for medical reasons. The doctors must tell you about any new information that might be important for you to allow your child to stay in the study. You have the right to ask for more information at any time. The doctor will respond to all your questions before you decide to enter the study

If you agree for your child to participate, you may provide a written consent. Or you may provide an oral consent along with your thumbprint and have a literate witness sign on your behalf.

*Whom to contact:*

You can ask any questions about this study or the consent form at any time. Also in case of any illness or if your child needs any medical attention during the study period, you can contact a doctor on the following telephone numbers:

24-hours telephone helpline number -

SMO: ______________________________ Contact number ________________

Medical Officer : _______________________Contact number ________________

If you have questions about your child’s participation in the study you may contact Mr/Ms/Dr. Chairman of the Institution Ethics Committee (IEC)

Name Phone Number

_________________________________ ________________________

**Certificate of Consent**

- I have been invited to have my child take part in the poliovirus vaccine trial in India.
- I have read the foregoing information, or it has been read to me. I understand the information I have received.
- I have had the opportunity to ask questions about it and any questions I have asked, have been answered to my satisfaction.
- I understand the benefits, discomforts, and risks of the study. I have the right to be notified of any new information that can be of importance to my child’s continuing in the study. If I have concerns, I can contact the doctor at any time.
- I consent voluntarily to have my child be a participant in this study and understand that I have the right to withdraw my child from the study at any time, without affecting my child’s medical care.

Print Name of Child’s parent

Print Signature of parent

Print Date / / (*dd/mm/yy*)

*If illiterate*

Print Name of Independent Literate Witness

Print Signature of Witness

Print Date / / (*dd/mm/yy*)

Affix thumb impression of parent

Print Name of Researcher

Print Signature of Researcher

Print Date / / (*dd/mm/yy*)

ANNEX IV

| **SUBJECT INFORMATION SHEET**  **For Study children in 10 - 11 years of age** |
| --- |

| **Study Title - Assessment of mucosal immunity to polioviruses after a supplemental dose of bivalent oral polio vaccine or inactivated poliovirus vaccine in children in Northern India: A Randomized Trial**  Protocol Number: Version Number:  **Sponsor: Panacea Biotec Limited** |
| --- |

**Please read the content carefully and ask questions from your doctor if any information is not clear. You can also ask questions or discuss regarding the study from your parent/guardian before saying yes for participation.**

**Why is this study being conducted?**

The information from this study would help to know the protection level of children in the area against polio virus infection and transmission. The study results will help the Government to understand the role of different vaccines in stopping the spread of this disease to achieve eradication of polio.

**Why you are being selected for study participation?**

In the study, we are including children in three different age groups (6-11 months, 5-6 years and 10-11 years) in this area and you qualify to participate on the inclusion criteria for the study

**How are you benefitted by participation in the study?**

You and your parents will know how well you are protected against polio. Also you will get more protection against polio through the additional dose/s of vaccine which will be given to you during the study. You will get a free medical check up done by a doctor during each visit in the study.

**What will you be required to do during the study?**

You will be enrolled in the study only if you fulfill the selection criteria and your parents or guardian agrres for you to be in the study. After they decide, you get to choose if you want to participate in the study. If you agree ……………………………..

- Your doctor will examine you.
- If eligible to participate, you will be expected to come to the health facility 4 times during the 2 month period.
- Your blood, saliva and stool samples will be taken at different intervals
- You will be given one or two doses of polio vaccine, either polio drops or the injection type vaccine
- You will be followed up for any study treatment related adverse event till satisfactory resolution or till last follow up visit, whichever is later..

**What is the risk involved for you?**

Vaccine administration and taking of samples will be done by qualified medical staff using safe and hygienic methods. Except little pain and redness at the site of needle prick, no other problems are anticipated.

**Will your information be disclosed to others?**

No, your name and participation will not be disclosed to others than the study staff.

**Your Participation in the Study and your Rights**

If you don’t want to be in the study, it is all right. If you start participation in the study now and want to discontinue later, you can do that without any effect on your right for treatment or services at this or other Government health facilities. If at any time during the study, you don’t feel well or suffer from any illness then please tell your parent/Guardian and/or the doctor. If you visit any other doctor for treatment, you need to inform that doctor that you are taking part in this study before he gives you any medicine/vaccine. Please avoid taking any medication without the knowledge of the study doctor. If you do, you should inform the doctor when you visit next time.

If your doctor feels this vaccine is not good for you, he/she may not give it next time. We will tell you if any new information becomes known during the study which may affect your willingness to continue your participation in the study.

Your doctor’s telephone number is [__________________________________]. You can call him/her if you have questions about the study or if you decide you don’t want to be in the study any more.

Parent /LAR Name: _________________________________

| **Assent Form**  **For Study children in 10 - 11 years of age**  **Study Title - Assessment of mucosal immunity to polioviruses after a supplemental dose of bivalent oral polio vaccine or inactivated poliovirus vaccine in children in Northern India: A Randomized Trial**  **Protocol Number: Version Number:**  **Sponsor: Panacea Biotec Limited** |
| --- |

Subject’s Name: _________________________________

Subject Initials: [__I__I__]

Subject Age (in Years-Months) [__I__]-[__I__] or

Date of Birth (DD-MM-YY): [__I__]-[__I__]-[__I__]

- I confirm that I have read and understood/have been explained the Subject information sheet for the above study and have had the opportunity to ask questions during the assent process for the study.
- I understand that my participation in the study is voluntary and that I am free to withdraw at any time, without giving any reason, without any medical care or legal rights being affected.
- I understand that authorities listed in the information sheet will not need my permission to look at my health records in respect of the current study.

**Note that you will only be included in this study if your parent/LAR also sign informed consent document for this study.**

I agree to take part in the above study.

| **Signature (or Thumb** ____________________________  **impression)of the Subject**  **Date:** [__I__]-[__I__]-[__I__]  Day Month Year  **Name of Subject:**__________________________________________ |
| --- |
| **Signature of person** ______________________________  **conducting assent**  **Date:** [__I__]-[__I__]-[__I__]  Day Month Year  **Name of person conducting assent:**______________________________ |
| **Signature of witness (if required)**______________________________  **(If required)**  **Date:** [__I__]-[__I__]-[__I__]  Day Month Year  **Name of witness:**__________________________________ |

ANNEX V. **Case report form and questionnaires**

**Mucosal immunity study in Moradabad**

**QUESTIONNAIRE – ENROLLMENT**

**Date ______ / _______ / ________**

# Identification data

# Child’s name:

# DOB: ____ \ _____ \ _____ Age: ______ years ______ months Sex F / M

Name of father:________________________________________________________________________

Address: Landmark:

# Village Block: CMC area

**Eligibility criteria**

- Child’s residence < 30 km from study site Yes / No
- Child’s age: 6-11 months, 61- 72 months or 120 – 131 months? Yes / No
- Child and family planning to travel / move in the next 2 months? Yes / No
- Diagnosis or suspicion of immunodeficiency in the child or family member? Yes / No
- Child has known coagulation disorders that contraindicate venipuncture? Yes / No
- Child has history of severe allergic reaction to a medication or to OPV? Yes / No
- Has the child had diarrhea in the last 1-2 days (3 or more liquid stools per day)? Yes / No
- Does the child have any acute infection or other acute condition now? Yes / No

If yes, specify _____________________________________

Temp: _________

**Based upon the above criteria is this child a candidate for the study? Yes / No**

***If child has any of the previous criteria that contraindicates participation, STOP THE QUESTIONNAIRE;*** *provide medical advice and oral medication if required, and say farewell.*

***If the child fulfills the enrollment criteria, EXPLAIN THE STUDY to the parents.***

**Parent/guardian’s response**

Accept participation Reject participation

***If parents REJECT to participate or NOT AVAILABLE*** *for the study duration, STOP and say farewell.*

***If parents ACCEPT to participate*** *arrange transportation to study site.*

**Name and signature of investigator:** ___________________________________________________

**Mucosal immunity study in Moradabad**

**Day 0 Procedures**

**Date _____ / _______ / _______ Child ID number:**

# Explanation and review of consent form

# Informed consent obtained? Yes / No

# Name and signature of person obtaining consent: ______________________________

# Epidemiology data

# DOB: ____ \ _____ \ _____ Age: ______ years ______ months Sex: F / M

Religion: H / M / O Father’s occupation:

Father’s education (encircle): Illiterate / Primary / Middle / Tenth / Twelfth / Graduate

Number of people in household (parents & siblings only): ­­

Number of children <5 years in household (study subject):

# Clinical exam

# Height: _________ cms Weight: ___________ kgs Temp: _____ F

**Sample collection**:

Blood collected? Yes / No Quantity sufficient (≥1ml)? Yes / No

If blood not collected, explain reasons:

Saliva sample collected? Yes / No

If saliva not collected, explain reasons:

Stool sample collected? Yes / No Date of collection ___ / ____ / ___

If stool not collected, explain reasons: _______________________________________________

Name and signature of responsible for sample collection:

**Vaccine administration**:

Type of vaccine administered: Intramuscular (IPV) Oral (bOPV) No vaccine

Lot and Batch number: Exp date: ___ / ___/ ___ Time of administration: ___ : ___

If intramuscular IPV, site of administration: Right arm Left arm

Observation for 30 minutes after vaccine administration:

Temp ________ Any symptoms: Yes / No.

If yes, describe symptoms: _______________________________________________________

If intramuscular IPV: Swelling: None <1 cm 1-3 cm > 3 cm

Redness: None <1 cm 1-3 cm > 3 cm

Name and signature of vaccinator _________________________________________

**Mucosal immunity study in Moradabad**

**Day 7 Procedures *(Only a subset of children)***

**Date _____ / _______ / _______ Child ID number:**

# Parent/Guardian agrees to attend the second study visit? Yes / No

# If no, specify reasons: _________________________________________________________________ ____________________________________________________________________________________

# Name and signature of person doing interview:

*If parents agree to the second visit, arrange transportation to the study site*

# Clinical exam

Child healthy? Yes / No *(If No, fill the adverse event form, and provide advice/referral)*

Since the last visit, - Has the child required hospitalization? Yes / No

- Has the child required a visit to a physician/clinic for health problems? Yes / No

If yes to previous hospitalization / physician visit, has the adverse event form been completed? Yes / No

Name and signature of investigator/physician:

**Sample collection**:

Blood collected? Yes / No Quantity sufficient (≥ 3 ml)? Yes / No

If blood not collected, explain reasons:

Name and signature of responsible for sample collection:

**Mucosal immunity study in Moradabad**

**Day 28 Procedures**

**Date _____ / _______ / _______ Child ID number:**

# Parent/Guardian agrees to attend the second study visit? Yes / No

# If no, specify reasons: _______________________________________________________________________________

# Name and signature of person doing interview:

*If parents agree to the second visit, arrange transportation to the study site*

# Clinical exam

Child healthy? Yes / No *(If No, fill the adverse event form, and provide advice/referral)*

Since the last visit, - Has the child required hospitalization? Yes / No

- Has the child required a visit to a physician/clinic for health problems? Yes / No

If yes to previous hospitalization / physician visit, has the adverse event form been completed? Yes / No

Name and signature of investigator/physician:

**Sample collection**:

Blood collected? Yes / No Quantity sufficient (≥ 3 ml)? Yes / No

If blood not collected, explain reasons:

Oral fluid sample collected? Yes / No

If oral fluid not collected, explain reasons:

Stool sample collected? Yes / No Date of collection ___ / ____ / ___

Quantity sufficient (≥ 10 g)? Yes / No

If stool not collected, explain reasons: _____________________________________________

Name and signature of responsible for sample collection:

**Administration of bOPV challenge dose**:

Oral bOPV administered? Yes / No Time of administration: (hh:mm) ___ : ____

If bOPV not administered, explain reasons: _______________________________________

Any problems within 15 minutes of administration (e.g. spitting, vomiting)?: Yes / No

Name and signature of vaccinator:

**Mucosal immunity study in Moradabad**

**Day 56 Procedures**

**Date _____ / _______ / _______ Child ID number:**

# Parent/Guardian agrees to attend the third study visit? Yes / No

# If no, specify reasons: __________________________________________________________________

# Name and signature of person doing interview:

*If parents agree to the second visit, arrange transportation to the study site*

# Clinical exam

Child healthy? Yes / No *(If No, fill the adverse event form, and provide advice/referral)*

Since the last visit, - Has the child required hospitalization? Yes / No

- Has the child required a visit to a physician/clinic for health problems? Yes / No

If yes to previous hospitalization / physician visit, has the adverse event form been completed? Yes / No

Name and signature of investigator/physician:

**Sample collection**:

Blood collected? Yes / No Quantity sufficient (≥ 3 ml)? Yes / No

If blood not collected, explain reasons:

Oral fluid sample collected? Yes / No

If oral fluid not collected, explain reasons:

Name and signature of responsible for sample collection:

**Mucosal immunity study in Moradabad**

**Day 31- Household stool sample collection**

**Date of visit _____ / _______ / _______ Child ID number:**

**Sample collection**:

Stool sample collected? Yes / No

If sample not collected, explain reasons:

*(Note: contact supervisory SMO for action if family not available at home or sample not collected for other reason. A second visit may be required for stool collection).*

Date when sample was collected _______ / ______ / ______

Quantity sufficient ((≥10 g)? Yes / No

Cold chain maintained? Yes / No

Name and signature of person responsible for stool collection:

**Mucosal immunity study in Moradabad**

**Day 35- Household stool sample collection**

**Date of visit _____ / _______ / _______ Child ID number:**

**Sample collection**:

Stool sample collected? Yes / No

If sample not collected, explain reasons:

(Note: contact supervisory SMO for action if family not available at home or sample not collected for other reason. A second visit may be required for stool collection).

Date when sample was collected _______ / ______ / ______

Quantity sufficient (≥10 g)? Yes / No

Cold chain maintained? Yes / No

Name and signature of person responsible for stool collection:

**Mucosal immunity study in Moradabad**

**Day 42- Household stool sample collection**

**Date of visit _____ / _______ / _______ Child ID number:**

**Sample collection**:

Stool sample collected? Yes / No

If sample not collected, explain reasons:

(Note: contact supervisory SMO for action if family not available at home or sample not collected for other reason. A second visit may be required for stool collection).

Date when sample was collected _______ / ______ / ______

Quantity sufficient (≥10 g)? Yes / No

Cold chain maintained? Yes / No

Name and signature of person responsible for stool collection:

**Mucosal immunity study in Moradabad**

**Adverse event report**

**Child ID number:**

# Date of report: ____ \ _____ \ _____ Name of SMO: _________________________________ Initials of legal guardian providing information: ________

Type of vaccine administered: Intramuscular (IPV) Oral (bOPV) No vaccine

Lot and Batch number: Exp date: ___ / ___/ ___

Date of administration: ____ / ____ / _____ Time of administration (hh:mm) ___ : ____ Study site:

# Description of the event

# Date when event started (dd / mm/yy): ____ / ____ / _____ Time (hh:mm): _____ : _____

# Describe symptoms and signs:

#

#

#

Actions taken: Medical advice Referral to health facility Referral to Hospital

If referred to health facility or Hospital, specify name:

Medications given:

Diagnostics tests performed:

Date when event resolved (dd / mm/yy): ____ / ____ / _____

Final diagnosis:

**Any of the following occurred or present?:**

Paralysis Anaphylaxis Hospitalization Death

(*If any of these are present, report to the study coordinator and AEFI Committee as a Serious Adverse Event)*

**To be filled by the AEFI committee**

Is the event related to the vaccine? Very likely Probable Possibly

Unlikely > Unrelated Unclassifiable

Comments by the AEFI:

ANNEX VI

# DECLARATION OF HELSINKI

**Ethical Principles for Medical Research Involving Human Subjects**

#### A. INTRODUCTION

1. The World Medical Association has developed the Declaration of Helsinki as a statement of ethical principles to provide guidance to physicians and other participants in medical research involving human subjects. Medical research involving human subjects includes research on identifiable human material or identifiable data.
2. It is the duty of the physician to promote and safeguard the health of the people. The physician's knowledge and conscience are dedicated to the fulfilment of this duty.
3. The Declaration of Geneva of the World Medical Association binds the physician with the words, "The health of my patient will be my first consideration," and the International Code of Medical Ethics declares that, "A physician shall act only in the patient's interest when providing medical care which might have the effect of weakening the physical and mental condition of the patient."
4. Medical progress is based on research, which ultimately must rest in part on experimentation involving human subjects.
5. In medical research on human subjects, considerations related to the well being of the human subject should take precedence over the interests of science and society.
6. The primary purpose of medical research involving human subjects is to improve prophylactic, diagnostic and therapeutic procedures and the understanding of the aetiology and pathogenesis of disease. Even the best-proven prophylactic, diagnostic, and therapeutic methods must continuously be challenged through research for their effectiveness, efficiency, accessibility and quality.
7. In current medical practice and in medical research, most prophylactic, diagnostic and therapeutic procedures involve risks and burdens.
8. Medical research is subject to ethical standards that promote respect for all human beings and protect their health and rights. Some research populations are vulnerable and need special protection. The particular needs of the economically and medically disadvantaged must be recognized. Special attention is also required for those who cannot give or refuse consent for themselves, for those who may be subject to giving consent under duress, for those who will not benefit personally from the research and for those for whom the research is combined with care.
9. Research Investigators should be aware of the ethical, legal and regulatory requirements for research on human subjects in their own countries as well as applicable international requirements. No national ethical, legal or regulatory requirement should be allowed to reduce or eliminate any of the protections for human subjects set forth in this Declaration.

##### B. BASIC PRINCIPLES FOR ALL MEDICAL RESEARCH

1. It is the duty of the physician in medical research to protect the life, health, privacy, and dignity of the human subject.
2. Medical research involving human subjects must conform to generally accepted scientific principles, be based on a thorough knowledge of the scientific literature, other relevant sources of information, and on adequate laboratory and, where appropriate, animal experimentation.
3. Appropriate caution must be exercised in the conduct of research, which may affect the environment, and the welfare of animals used for research must be respected.
4. The design and performance of each experimental procedure involving human subjects should be clearly formulated in an experimental protocol. This protocol should be submitted for consideration, comment, guidance, and where appropriate, approval to a specially appointed ethical review committee, which must be independent of the investigator, the sponsor or any other kind of undue influence. This independent committee should be in conformity with the laws and regulations of the country in which the research experiment is performed. The committee has the right to monitor ongoing trials. The researcher has the obligation to provide monitoring information to the committee, especially any serious adverse events. The researcher should also submit to the committee, for review, information regarding funding, sponsors, institutional affiliations, other potential conflicts of interest and incentives for subjects.
5. The research protocol should always contain a statement of the ethical considerations involved and should indicate that there is compliance with the principles enunciated in this Declaration.
6. Medical research involving human subjects should be conducted only by scientifically qualified persons and under the supervision of a clinically competent medical person. The responsibility for the human subject must always rest with a medically qualified person and never rest on the subject of the research, even though the subject has given consent.
7. Every medical research project involving human subjects should be preceded by careful assessment of predictable risks and burdens in comparison with foreseeable benefits to the subject or to others. This does not preclude the participation of healthy volunteers in medical research. The design of all studies should be publicly available.
8. Physicians should abstain from engaging in research projects involving human subjects unless they are confident that the risks involved have been adequately assessed and can be satisfactorily managed. Physicians should cease any investigation if the risks are found to outweigh the potential benefits or if there is conclusive proof of positive and beneficial results.
9. Medical research involving human subjects should only be conducted if the importance of the objective outweighs the inherent risks and burdens to the subject. This is especially important when the human subjects are healthy volunteers.
10. Medical research is only justified if there is a reasonable likelihood that the populations in which the research is carried out stand to benefit from the results of the research.
11. The subjects must be volunteers and informed participants in the research project.
12. The right of research subjects to safeguard their integrity must always be respected. Every precaution should be taken to respect the privacy of the subject, the confidentiality of the patient's information and to minimize the impact of the study on the subject's physical and mental integrity and on the personality of the subject.
13. In any research on human beings, each potential subject must be adequately informed of the aims, methods, sources of funding, any possible conflicts of interest, institutional affiliations of the researcher, the anticipated benefits and potential risks of the study and the discomfort it may entail. The subject should be informed of the right to abstain from participation in the study or to withdraw consent to participate at any time without reprisal. After ensuring that the subject has understood the information, the physician should then obtain the subject's freely given informed consent, preferably in writing. If the consent cannot be obtained in writing, the non-written consent must be formally documented and witnessed.
14. When obtaining informed consent for the research project the physician should be particularly cautious if the subject is in a dependent relationship with the physician or may consent under duress. In that case the informed consent should be obtained by a well-informed physician who is not engaged in the investigation and who is completely independent of this relationship.
15. For a research subject who is legally incompetent, physically or mentally incapable of giving consent or is a legally incompetent minor, the investigator must obtain informed consent from the legally authorized representative in accordance with applicable law. These groups should not be included in research unless the research is necessary to promote the health of the population represented and this research cannot instead be performed on legally competent persons.
16. When a subject deemed legally incompetent, such as a minor child, is able to give assent to decisions about participation in research, the investigator must obtain that assent in addition to the consent of the legally authorized representative.
17. Research on individuals from whom it is not possible to obtain consent, including proxy or advance consent, should be done only if the physical/mental condition that prevents obtaining informed consent is a necessary characteristic of the research population. The specific reasons for involving research subjects with a condition that renders them unable to give informed consent should be stated in the experimental protocol for consideration and approval of the review committee. The protocol should state that consent to remain in the research should be obtained as soon as possible from the individual or a legally authorized surrogate.
18. Both authors and publishers have ethical obligations. In publication of the results of research, the investigators are obliged to preserve the accuracy of the results. Negative as well as positive results should be published or otherwise publicly available. Sources of funding, institutional affiliations and any possible conflicts of interest should be declared in the publication. Reports of experimentation not in accordance with the principles laid down in this Declaration should not be accepted for publication.

**C. ADDITIONAL PRINCIPLES FOR MEDICAL RESEARCH COMBINED WITH MEDICAL CARE**

1. The physician may combine medical research with medical care, only to the extent that the research is justified by its potential prophylactic, diagnostic or therapeutic value. When medical research is combined with medical care, additional standards apply to protect the patients who are research subjects.
2. The benefits, risks, burdens and effectiveness of a new method should be tested against those of the best current prophylactic, diagnostic, and therapeutic methods. This does not exclude the use of placebo, or no treatment, in studies where no proven prophylactic, diagnostic or therapeutic method exists.^1^
3. At the conclusion of the study, every patient entered into the study should be assured of access to the best-proven prophylactic, diagnostic and therapeutic methods identified by the study.^2^
4. The physician should fully inform the patient which aspects of the care are related to the research. The refusal of a patient to participate in a study must never interfere with the patient-physician relationship.
5. In the treatment of a patient, where proven prophylactic, diagnostic and therapeutic methods do not exist or have been ineffective, the physician, with informed consent from the patient, must be free to use unproven or new prophylactic, diagnostic and therapeutic measures, if in the physician's judgement it offers hope of saving life, re-establishing health or alleviating suffering. Where possible, these measures should be made the object of research, designed to evaluate their safety and efficacy. In all cases, new information should be recorded and, where appropriate, published. The other relevant guidelines of this Declaration should be followed**.**

^1^ **Note of clarification on paragraph 29 of the WMA Declaration of Helsinki**

The WMA hereby reaffirms its position that extreme care must be taken in making use of a placebo-controlled trial and that in general this methodology should only be used in the absence of existing proven therapy. However, a placebo-controlled trial may be ethically acceptable, even if proven therapy is available, under the following circumstances:

- Where for compelling and scientifically sound methodological reasons its use is necessary to determine the efficacy or safety of a prophylactic, diagnostic or therapeutic method; or

- Where a prophylactic, diagnostic or therapeutic method is being investigated for a minor condition and the patients who receive placebo will not be subject to any additional risk of serious or irreversible harm.

All other provisions of the Declaration of Helsinki must be adhered to, especially the need for appropriate ethical and scientific review.

^2^ **Note of clarification on paragraph 30 of the WMA Declaration of Helsinki**

The WMA hereby reaffirms its position that it is necessary during the study planning process to identify post-trial access by study participants to prophylactic, diagnostic and therapeutic procedures identified as beneficial in the study or access to other appropriate care. Post-trial access arrangements or other care must be described in the study protocol so the ethical review committee may consider such arrangements during its review.

ANNEX VII

Poliovirus Serology

Microneutralization Test for Polio Antibodies

| **Prepared by** | **Date Adopted** | **Supersedes Procedure** |
| --- | --- | --- |
| N. Dybdahl-Sissoko | 2002 | Polio Lab Manual |

| **Review Date** | **Revision Date** | **Lab Supervisor** | **CDC CLIA Director** |
| --- | --- | --- | --- |
| 8/2/04 | 8/2/04 |  |  |

#### Introduction

This is a test to measure neutralizing antibody titers to poliovirus types 1, 2, and 3 using a modified microneutralization assay. If more than 7 sera are being tested, the samples must be randomized using a balanced block randomization scheme with integrated controls. As described below, up to 100 sera may be tested per run. The test requires an incubation period of 5 days; therefore it is best to run the test on Wednesday, Thursday, or Friday so that the plates can be stained on Monday, Tuesday, or Wednesday. Up to 100 sera, which fit on 79 plates, may be tested per day.

#### Materials and Equipment

- Hep-2C cells
- T-150 tissue culture flasks
- 96-well tissue culture plates
- Plastic wrap
- Microcentrifuge tubes (Marsh 1.7ml)
- Pipettes; 10ml, 25ml
- Pipettors: p20-200, 12-channel p20-200, repeater pipette p20-200
- Pipette tips (Rainin)
- Pipette tips for Cetus
- EMEM (SRP Cat.# CP0047)
- Hyclone FBS (SRP Cat.# CP0039)
- Tween-20
- In House Reference Sera (REVB/EVS freezer #60)
- Sabin virus stocks grown in Hep2C cells (REVB/EVS freezer #60)
- 37°C incubator
- Cetus Pro/Pette System (automatic dilutor)
- Elisa plate washer
- Elisa plate reader
- Crystal violet stain

Mix together and let sit until dissolved (may be stored up to one year):

2g crystal violet

1000ml 95% ethanol

For Use:

500ml crystal violet/ethanol mixture

10ml Tween-20

1500ml deionized H_2_0

**Preparation**

In the week or two preceding the test runs:

1. Assign sera randomly to each run using a balanced block randomization scheme.
2. Use the list generated by the randomization to label plates. This protocol has a diagram of the plate set-up. Each test serum is run in triplicate; so 4 sera may be put on each plate. Each plate is duplicated two more times for the other 2 poliovirus serotypes. There will be a back-titration plate for each serotype, and one cell control plate.

| RUN | P1 Plate | P2 Plate | P3 Plate | Position | DASH # |
| --- | --- | --- | --- | --- | --- |
| 420 | 5 | 22 | 39 | 1 | 99010332 |
| 420 | 5 | 22 | 39 | 2 | 99010327 |
| 420 | 5 | 22 | 39 | 3 | 99010362 |
| **420** | **5** | **22** | **39** | **4** | **99010370** |
| 420 | 6 | 23 | 40 | 1 | 99010376 |
| 420 | 6 | 23 | 40 | 2 | 99010341 |
| 420 | 6 | 23 | 40 | 3 | 99010378 |
| 420 | 6 | 23 | 40 | 4 | 99010371 |
| 420 | 7 | 24 | 41 | 1 | 99010348 |
| **420** | **7** | **24** | **41** | **2** | **387001** |
| 420 | 7 | 24 | 41 | 3 | 99010363 |
| 420 | 7 | 24 | 41 | 4 | 99010383 |

1. In this example of a randomized sample list, serum sample number 99010370 will be in run number 420, position 4 on plates 5 (P1), 22 (P2), and 39 (P3). The IRS is in position 2 on plates 7 (P1), 24 (P2), and 41 (P3).
2. Label caps of 1.7ml microcentrifuge tubes with last 4 digits of the DASH number in the order generated by the randomized list.
3. Prepare MEM 2% FBS (add 20ml fetal bovine serum to 1000 ml bottle of EMEM) Serum must be inactivated at 56°C for 30 minutes and filtered with a Nalgene filter 450-0020.

Tuesday prior to each test run:

Seed T-150 flasks with 30ml of Hep2-C cells at 5x10^5^ cells/ml. Cells must be ordered at least 10 days and up to two months ahead of time through the SRP website: https://srpapps.cdc.gov. The CDC Cell Culture Development Team prepares cell suspensions at a concentration of 5 x 10^5^ cells/ml. Seed enough flasks to have ~1 or 2 flasks for 20 plates.

One day before test run:

1. Aliquot 70 µl test sera into pre-numbered microcentrifuge tubes and inactivate at 56°C for 30 minutes, store at 4°C.
2. Thaw IRS (In House Reference Serum) and dilute 1:8. (IRS has been previously inactivated at 56°C for 30 minutes and is stored at -70°C)
   - 280µl aliquots - add 1960 µl MEM 2% FBS day of test
   - 375µl aliquots - add 2625 µl MEM 2% FBS day of test

Day of test run:

1. Add 490µl MEM 2% FBS to 70µl heat inactivated test sera aliquots (for a final dilution of 1:8).
2. Add 25µl MEM 2% FBS to rows B-H of the test plates with multichannel pipette.
3. Add 25µl MEM 2% FBS to each row of back titration plates (S1, S2 and S3).
4. Add 50µl MEM 2% FBS to every well of the cell control plate.
5. Add 50µl MEM 2% FBS of each serum dilution (1:8) to row “A” only of the test serum plate (3 well/serum dilution and 4 test sera per plate – see figure).
6. Repeat this procedure for the poliovirus type 2 and type 3 test serum plates.
7. Make serial 2-fold dilutions with Cetus dilutor from row A through row H (serum dilutions will range from 1:8 to 1:1024).
8. Dilute each poliovirus serotype in MEM 2% to contain 100 TCID_50_ according to figure below. Prepare sufficient virus challenge suspension for the number of sera to be tested; each plate requires approximately 2.5ml of diluted challenge virus.
9. Prepare the back titrations of each poliovirus serotype in MEM 2% FBS. Titrate each virus from 100 TCID_50_ a further three 10-fold steps. (see figure below)
10. 10.

| **Virus Stock** In Hep2-C | Aliquoted 08/09/99 | | | 1000µl/vial | |  |  |
| --- | --- | --- | --- | --- | --- | --- | --- |
| **Sabin 1** 100 TCID_50_ = 10^-5.2^ |  |  | | 100 | 10 | 1 | 0.1 |
| virus 633µl 100µl | 100µl | 100µl | 800µl  5.4ml 45ml | 7ml | 100µl | 100µl | 100µl |
| medium 367µl 900µl | 900µl | 900µl | 7.2ml | 63ml | 900µl | 900µl | 900µl |
| **Sabin 2** 100 TCID_50_ = 10^-5.35^ |  |  |  |  |  |  |  |
| virus 448µl 100µl | 1004µl | 100µl | 800µl  5.4ml 45ml | 7ml | 100µl | 100µl | 100µl  f |
| medium 552µl 900µl | 900µl | 900µl | 7.2ml | 63ml | 900µl | 900µl | 900µl |
| **Sabin 3** 100 TCID_50_ =10^-5.0^ |  |  |  |  |  |  |  |
| virus 100µl | 100µl | 100µl | 800µl  5.4ml 45ml | 7ml | 100µl | 100µl | 100µl  _ |
| medium 900µl | 900µl | 900µl | 7.2ml | 63ml | 900µl | 900µl | 900µl |

1. Add 25µl of 100 TCID_50_ of relevant poliovirus antigen to all wells in the test serum plates.
2. To the back titration plates:

- Add 25µl of 100 TCID_50_ of virus to rows A and B (*i.e*., 24 wells/dilution)
- Similarly, add 25µl of the next three 10-fold dilutions to rows C and D, E and F, and G and H, respectively.

1. Wrap all plates in plastic wrap and tap gently to mix. Incubate for 3 hours at 36°C and 5% CO_2_.
2. During incubation, wash Hep2-C monolayer cell cultures, trypsinize, centrifuge, count cells, and prepare a cell suspension in MEM 10% to contain approximately 3 x 10^5^ cells/ml. Prepare a sufficient volume of cells; each plate requires approximately 2.5 ml of cell suspension, and every 20 plates requires 1 to 2 T-150 of confluent cells. Store cells in glass bottle at 4°C until ready to use.
3. Add 25µl of prepared cell suspension to each well of every plate.
4. Wrap all plates in plastic wrap and tap gently to mix. Incubate for 5 days at 36°C and 5% CO_2_

**Staining Plates**

1. After 5 days incubation, stain plates with crystal violet solution.
2. Carefully, so as not to disturb cell monolayer, use Elisa plate washer to aspirate medium completely from wells.
3. With multichannel pipette, add crystal violet solution so that each well is half full (~100µl).
4. Incubate plates 40 minutes under hood.
5. Use Elisa plate washer to wash each plate 3 times with tap water.
6. Allow plates to dry under the hood overnight.
7. Read plates with Tecan Spectrafluor Elisa Reader at 570 nm wavelength.
8. Plates should be kept at room temperature until results are calculated.

**Reading plates**

1. Turn on power to the Tecan SPECTRAFluor Plus spectrophotometer and to the computer.
2. Double click the shortcut to XFluor4 icon.
3. Click “Enable Macros”.
4. Click on “Xfluor4" on the tool bar and select “connect”.
5. Select “ok” for Setup Port.
6. Click XFluor4 on the tool bar and select “Edit Measurement Parameter”.
7. Select “Absorbance”.
8. Click the tab “Meas. Params” and check that the filter is set for 570 nm and click “ok”.
9. Put first plate on tray, click XFluor4 on the tool bar and select “Start Measurement”.
10. Once the plate has been read, remove plate.
11. Add next plate and repeat step 11-12.

**ANNEX VIII:**

Antibody-Capture ELISA for IgA and IgM to Polioviruses Type 1, 2, and 3

**1. Introduction**

IgA and IgM antibody detection in serum specimens from children previously immunized with either inactivated poliovirus vaccine (IPV) or oral poliovirus vaccine (OPV) is necessary to determine a child’s immunization status in a post eradication era.

**ELISA test**: Wells of microtiter plates are coated with either goat anti-human IgA or goat anti-human IgM antibodies. After washing, wells are blocked with gelatinand human serum samples are applied and allowed to bind to the coating antibody. After washing off any unbound human sera, type-specific antigen is applied and allowed to bind to the serum antibodies. After washing off unbound antigen, type-specific mouse monoclonal antibody is added to detect bound antigen. After washing off unbound monoclonal antibody, peroxidase-labeled anti-mouse conjugate is added to detect bound type specific monoclonal antibody. After washing off unbound conjugate, the captured conjugate is then measured using a detection system employing TMB. The final reaction is stopped by adding a specific citric acid solution. Plates are then scanned by a spectrophotometer at 620nm and calculations are performed to determine the result.

**2. Reagents**

a. Carbonate buffer: 0.05M, pH 9.6, plus azide. CDC product I.D.: 0121A

b. Wash buffer: PBS: phosphate-buffered saline (0.01 M) pH 7.2 with Tween-20 (0.05%), CDC product I.D.: 4585.

c. Blocking and dilution buffer: P-G-T, PBS (0.01M) pH 7.2, 0.5% gelatin, 0.15% Tween-20. PBS (0.01 M, pH 7.2), CDC product I.D.: 4539, Difco Gelatin, Fisher cat. No:DF0143-17-9, Polyoxyelthylene- Sorbitan Monolaurate, Tween-20, Sigma cat. No: P-1379.

d. Capture antibodies: These products are commercially available.

Affinity-purified antibody, anti-human IgA (α), KPL, Inc. catalog No: 01-10-

01

Affinity-purified Antibody, anti-human IgM (µ), KPL, Inc. catalog No: 01-

10-03

e. Sera: Human serum, minimum volume for IgA and IgM is 7ul diluted

1:200.

f. Antigens: (RIVM) Rijksinstituut voor Volksgezondheid en Milieu (National Institute for Public Health and the Environment), and (NIBSC) National Institute for Biological Standards and Control.

IPV1: RIVM inactivated poliovirus type-1, Pu96-1285-907.

IPV2: RIVM inactivated poliovirus type-2, Pu97-273-907.

IPV3: RIVM inactivated poliovirus type-3, Pu05-3454-907.

Sabin 1: NIBSC 01/528, RD2, 2/10/2009.

Sabin 2: NIBSC 01/530, RD2, 2/10/2009.

Sabin 3: NIBSC 01/532, RD2, 2/10/2009.

g. Monoclonal antibodies: These products are commercially available

Antibody Shop, anti-poliovirus type-1, IPV, HYB 295-17-02.

Antibody Shop, anti-poliovirus type-2, IPV, HYB 294-06.

Antibody Shop, anti-poliovirus type-3, IPV, HYB 300-06.

Millipore, poliovirus type-1, Sabin, 36542P.

Millipore, poliovirus type-2, Sabin, 36543P.

Millipore, poliovirus type-3, Sabin, 36544P.

h. Detector antibody: This product is commercially available.

KPL, Inc., goat anti-mouse IgG (H&L), human sera adsorbed, horseradish

peroxidase labeled, KPL catalog No. 074-1806.

i. Substrate: This product is commercially available.

KPL, Inc., SureBlue ReserveTM TMB peroxidase substrate. 3,3',5,5' Tetramethylbenzidine, KPL catalog No. 53-00-02.

j. Stop solution: This product is commercially available.

KPL, Inc. TMB BlueStoptm solution, citric acid, KPL catalog No. 50-85-30.

**3. Equipment and Supplies:**

a. Immulon 2HB, 96-well polystyrene, flat-bottom, high-binding. Thermo No: 3455 (Fisher cat . no: 14-245-61)

b. Bio-safety cabinet (Baker, Sterilguard III)

c. Incubator (Precision)

d. Multichannel Pipette (Gilson 50-200 ul)

e. Pipettes: (Gilson LTS 2ul, 10 ul, 20 ul, 200 ul, 1000 ul)

f. Deep well dilution racks (VWR)

g. 15 ml and 50 ml centrifuge tubes (Falcon)

h. 2 ml, 5 ml, 10 ml, and 25 ml serological pipettes (Falcon)

i. Reagent reservoirs (Costar)

j. Scale (Ohaus, Adventurer)

k. Stir plate (Thermolyne)

l. Freezers at -20oC and -70oC

m. Refrigerator +4oC

n. ELISA washer (SKATRON, ScanWasher300)

o. ELISA reader (TECAN SPECTRAFluor Plus spectrophotometer)

4. Procedure:

Coat the capture antibody onto the solid phase of a microplate

a. Using a multichannel pipette, coat labeled 96-well microplates with 50 ul/well of a 1:500 dilution of goat anti-human IgA in Carbonate Buffer. (50ug/well)

b. Using a multichannel pipette, coat labeled 96-well microplates with 50 ul/well of a 1:500 dilution of goat anti-human IgM in Carbonate Buffer. (50ug/well)

c. Incubate the microplates for 60 minutes, 37oC (plates flat in moist chamber,not stacked).

d. Wash the plates 4X with wash buffer. (Tap plates on a paper towel to remove residual liquid).

Addition of blocking buffer

e. Using a multichannel pipette, add 200 ul of P-G-T/well.

f. Incubate the microplates for 60 minutes, 37oC (plates flat in moist chamber, not stacked).

g. Wash the plates 4X with wash buffer. (Tap plates on a paper towel to

remove residual liquid).

Addition of serum

h. Using a multichannel or single-channel pipette, add 50 ul of a 1:200 dilution in P-G-T of each positive-control serum, negative-control serum, and test serum, vertically across the microplate to the appropriate wells. (Test serum in columns 1 and 2, 3 and 4, 5 and 6, 7 and 8, 9 and 10, 11and 12).

i. Incubate the microplates for 90 minutes, 37oC (plates flat in moist chamber,

not stacked).

j. Aspirate serum, fill wells with wash buffer and allow plates to soak for 3 minutes. Aspirate wash buffer, fill wells with wash buffer and allow plates to soak for an additional 3 minutes. Wash the plates 4X with wash buffer. (Tap plates on a paper towel to remove residual liquid).

Addition of type specific antigen

k. Using a multichannel pipette, add 50 ul of a 1:20 dilution in P-G-T of each antigen to the appropriate plates and wells. (Antigen in columns 1-12, rows A-H).

l. incubate the microplates overnight at room temperature (plates flat in moist chamber, not stacked).

m. As pirate serum, fill wells with wash buffer and allow plates to soak for 3 minutes. Aspirate wash buffer, fill wells with wash buffer and allow plates to soak for an additional 3 minutes. Wash the plates 4X with wash buffer. (Tap plates on a paper towel to remove residual liquid).

Addition of type specific monoclonal antibodies

n. Using a multichannel pipette, add 50 ul of a 1:40000 dilution in P-G-T of each MoAb to the appropriate plates and wells. (MoAb in columns 1-12,rows A-H).

o. Incubate the microplates for 90 minutes, 37oC (plates flat in moist chamber, not stacked).

p. As pirate serum, fill wells with wash buffer and allow plates to soak for 3 minutes. Aspirate wash buffer, fill wells with wash buffer and allow plates to soak for an additional 3 minutes. Wash the plates 4X with wash buffer. (Tap plates on a paper towel to remove residual liquid).

Addition of horseradish peroxidase labeled conjugate

q. Using a multichannel pipette, add 50 ul of a 1:5000 dilution in P-G-T of goat anti-mouse IgG (H&L) horseradish peroxidase labeled conjugate. (HRP conjugate in columns 1-12, rows A-H))

r. Incubate the microplates for 90 minutes, 37oC (plates flat in moist chamber, not stacked).

s. Aspirate serum, fill wells with wash buffer and allow plates to soak for 3 minutes. Aspirate wash buffer, fill wells with wash buffer and allow plates to soak for an additional 3 minutes. Wash the plates 4X with wash buffer. (Tap plates on a paper towel to remove residual liquid).

Addition of SureBlue ReserveTM Substrate

t. Using a multichannel pipette, add 50 ul of SureBlue ReserveTM Substrate previously warmed to room temperature. (Substrate in columns 1-12, rows A-H).

u. Incubate the microplates for 15 minutes undisturbed at room temperature.(plates flat, not stacked).

Addition of TMB BlueStopTM Solution

v. Using a multichannel pipette, add 50 ul of TMB BlueStopTM Solution previously warmed to room temperature. (BlueStopTM in columns 1-12 rows A-H).

w. Read absorbance of each plate 10 minutes post-addition of the BlueStopTM Solution using a 620 nm filter.

Figure 1:

|  | 1 | 2 | 3 | 4 | 5 | 6 | 7 | 8 | 9 | 10 | 11 | 12 |
| --- | --- | --- | --- | --- | --- | --- | --- | --- | --- | --- | --- | --- |
| A | Pos. Control | Pos. Control | Spec. 6 | Spec. 6 | Spec. 14 | Spec. 14 | Spec. 22 | Spec. 22 | Spec. 30 | Spec. 30 | Spec. 38 | Spec. 38 |
| B | Neg. Control | Neg. Control | Spec. 7 | Spec. 7 | Spec. 15 | Spec. 15 | Spec. 23 | Spec. 23 | Spec. 31 | Spec. 31 | Spec. 39 | Spec. 39 |
| C | N/S | N/S | Spec. 8 | Spec. 8 | Spec. 16 | Spec. 16 | Spec. 24 | Spec. 24 | Spec. 32 | Spec. 32 | Spec. 40 | Spec. 40 |
| D | Spec.  1 | Spec.  1 | Spec. 9 | Spec. 9 | Spec. 17 | Spec. 17 | Spec. 25 | Spec. 25 | Spec. 33 | Spec. 33 | Spec. 41 | Spec. 41 |
| E | Spec.  2 | Spec.  2 | Spec. 10 | Spec. 10 | Spec. 18 | Spec. 18 | Spec. 26 | Spec. 26 | Spec. 34 | Spec. 34 | Spec. 42 | Spec. 42 |
| F | Spec.  3 | Spec.  3 | Spec. 11 | Spec. 11 | Spec. 19 | Spec. 19 | Spec. 27 | Spec. 27 | Spec. 35 | Spec. 35 | Spec. 43 | Spec. 43 |
| G | Spec.  4 | Spec.  4 | Spec. 12 | Spec. 12 | Spec. 20 | Spec. 20 | Spec. 28 | Spec. 28 | Spec. 36 | Spec. 36 | Spec. 44 | Spec. 44 |
| H | Spec.  5 | Spec.  5 | Spec. 13 | Spec. 13 | Spec. 21 | Spec. 21 | Spec. 29 | Spec. 29 | Spec. 37 | Spec. 37 | Spec. 45 | Spec. 45 |

**ANNEX IX**

**IVI Laboratory**

Standard Operating Procedure –(SOP--)

**ELISPOT for enumeration of Polio-specificIgG and IgA antibody secreting cells**

**(Draft)**

# PURPOSE

The purpose of this SOP is to describe the procedure for performing the one step, dual color Enzyme Linked ImmunoSPOT assay (ELISPOT) for enumeration of IgG and IgA Antibody Secreting Cells (ASC)

1. **SCOPE**

The procedure outlined in this SOP will be applied to human peripheral blood mononuclear cells for use in Enzyme Linked ImmunoSPOT assay (ELISPOT) for detection and quantification of IgG and IgA ASC in subjects receiving a Polio vaccine.

**Abbreviations**

ASC: antibody secreting cells

HRP: Horse radish peroxidase

AP: Alkaline phospahatase

AEC: 3-Amino-9-Ethyl-carbazole

BCIP/NBT: 5-Bromo-4-chloro-3-indolyl phosphate ρ-toluidine salt/ Nitroblue tetrazolium

chloride

BSA: Bovine Albumin Fraction V

°C: Degrees Celsius

µl: Microlitre

ml: Millilitre

**PBS: Phosphate-Buffered Saline**

**PAGE OF CHANGES**

| Date of change/  date of draft: | Version-number | Changed page(s): | Summary of the change(s): | Changed by/ Sign |
| --- | --- | --- | --- | --- |
|  |  |  |  |  |

# MATERIALS AND EQUIPMENT

1. Gloves (latex, vinyl or nitrile).
2. Cool box and cool packs
3. Vortex
4. Parafilm
5. Single (10, 20, 200, 1000 ul) and multichannel pipette (5-50 ul, 30-300 ul)
6. Sterile pipette tips of various volume
7. Pipette aid and serological pipettes (5, 10, 25 ml)
8. Reagent reservoir (Corning # 4870 or equivalent)
9. Sterile polypropylene conical-bottom tubes (50 ml and 15 ml)
10. Millipore Multiscreen-HA 96-well plates, non-sterile. (Millipore #MSHA N4510)
11. Sterile Calcium and Magnesium-free PBS (GIBCO #10010-023 or equivalent).
12. ELISpot medium. Follow SOP-- Media, buffers and substrates preparation
13. Washing Buffer. Follow SOP-- Media, buffers and substrates preparation
14. Purified Goat anti-human lambda (Southern Biotech # 2070-01, Batch n.--,1 mg/ml). Store at 4˚C (Refrigerator n.).
15. Purified Goat anti-human kappa (Southern Biotech # 2060-01, Batch n --,1 mg/ml). Store

at 4˚C (Refrigerator n.).

1. Goat anti-human IgG (γ-chain specific) HRP-conjugated Antibody (Southern Biotechnology # 2040-05, 1 ml). Store at 4˚C (Refrigerator n.).
2. Goat anti-human IgA (α-chain specific) AP-conjugated Antibody (Southern Biotechnology # 2050-04, 1 ml). Store at 4˚C (Refrigerator n.).
3. Plate washer 12 channel (ImmunoWasher, NUNC # 470175). Adjust the vacuum pressure in order to avoid membrane damages.
4. BCIP/NBT liquid substrate ready to use (Sigma #B-1911, 100 ml) for AP-conjugated antibody. Follow SOP-- Media, buffers and substrates preparation
5. AEC substrate for HRP conjugated antibody. Follow SOP--Media, buffers and substrates preparation
6. Portable Incubator (Millipore)
7. 20 ml syringe.
8. Syringe Filter 0.45 µm (Millipore #SLHV 033 RS)
9. Polio Antigens (50 µg/ml)
   1. **PROCEDURE**
10. Coat wells with 100ul of each Polio antigen (c.a.1-5 ug/ml) –monovalent inactivated poliovirus type 1, 2 or 3) or κ/λ (each, 7 ug/ml) (refer to layout in appendix 1) in PBS
11. Non-coated wells are filled with PBS
12. Wrap the plate with parafilm and incubate overnight at 2-8°C
13. Discard the coating antigen by flicking the plate
14. Wash plate three times with 200 µl of PBS
15. Add 200 μl/well of ELIspot medium (refer to SOP—Media, buffers and substrates preparation) and incubate in portable incubator at 37°C for ≥ 30 minutes.
16. Using 1 ml pipette and sterile tip, transfer 1 ml of heparin anti-coagulated blood from Vacutainer® to a labeled 15 ml tube.
17. Centrifuge at 600 g x 10 min at 20 °C or allow to sediment at 4-8°C x 60 min.
18. Remove plasma using 1 ml tip. Note: Do not remove the blood.
19. Resuspend the remaining fraction by tapping the bottom of the tube
20. Add 9 ml of RBC Lysing buffer containing 25ul of α4β7^+^ or HLA-DR/CD19^+^ beads per ml of blood (refer to SO-- Media, buffers and substrates preparation, Preparation of the α4β7^+^ or HLA-DR/CD19^+^ coated beads).
21. Invert the tube and resuspend the pellet by tapping the bottom of the tube. Note: make sure that blood is well resuspended and no pellet is present in the bottom of the tube
22. Incubate for 15 min at room temperature. Invert the tube twice every 2 min.
23. Apply the magnet for 4 min.
24. Without detaching the magnet, remove supernatant (lysis buffer containing non captured cells and red cells ghosts) with a 5 ml sterile pipette. Note: do not touch with the pipette the wall of the tube containing the beads.
25. Without detaching the magnet, add 10 ml of cell isolation buffer
26. Without detaching the magnet, remove cell isolation buffer
27. Repeat Step 16.
28. Remove cell isolation buffer and remove the tube form the magnet.
29. Resuspend the cells-beads fraction in ELISpot medium (1 tube=1 ml of whole blood equivalent is resupended in 500 µl of ELISpot medium).
30. Decant Blocking solution from Elispot wells by flicking the plate. Note: Make sure that only a minimal amount of liquid is left. Don’t let the membrane dry, add immediately the diluted antibody (see step 22).
31. Add immediately 50 µl of diluted antibodies to each well of the plate using appropriate multi-channel and tips. Diluted antibodies : mixture of anti-human IgG-HRP (dilution 1:500; final dilution 1:1000) and anti-human IgA-AP conjugated antibodies (1:375; final dilution 1:750)
32. Transfer 50 µl of cells-beads into the ELISPOT plate
33. Incubate the plate at 37°C for 3 hours. Note: Do not move or shake the incubator during this time.
34. Wash plates with PBS seven times and with PBST three times.
35. Prepare the BCIP/NBT substrate by filtering with 0.45 um syringe filter.
36. Wash plates with distilled water five times.
37. Remove the excess of water tapping both side of the plate with tissue paper

Note: Don’t let the membrane dry.

1. Add 80 µl of BCIP/NBT substrate for each well of the plates using appropriate multi-channel and tips. Keep plates out of direct light.
2. Incubate for 20-30 minutes at room temperature in the dark
3. Prepare AEC substrate “ready to use” (refer to SOP-- Media, buffers and substrates preparation)
4. Discard the BCIP/NBT substrate by flicking the plate.
5. Repeat steps 34 and 35.
6. Add 100 µl of AEC substrate for each well of the plates using appropriate multi-channel and tips. Let reaction proceed for 20-30 minutes.
7. Discard the AEC substrate by flicking the plate.
8. Carefully remove the under drain (the plastic support under the plate) and rinse the membrane (front and back) with distilled water (extensively).
9. Let the membrane dry before read it, either by standing the plate in up-side down position at room temperature.
10. Count the number of spots for each Igs isotype (IgG red and IgA blue) in each well using an automated reader or a portable stereo microscope (magnification x 6).
11. Store the plate in the dark at room temperature.

**Appendix 1. Layout of the ELISPOT plate**

Coat plates with 100ul of each Polio-antigens (c.a.1-5 ug/ml) and BL are coated with 100 ul of PBS.

Coat plates with 100ul of κ/λ (each, 7 ug/ml) and BL are coated with 100 ul of PBS.
